# An Efficient and High-Accuracy Modeling and Design Method for High-Load, Large-Stroke Piezo-Actuated Compliant Amplification Mechanisms

**DOI:** 10.3390/mi17091004

**Published:** 2026-08-25

**Authors:** Houwen Fu, Wenxi Zhang, Ziqi Wang, Boying Qiao, Hongke Wang

**Affiliations:** 1 Aerospace Information Research Institute, Chinese Academy of Sciences, Beijing 100094, China; fuhw185@gmail.com (H.F.);; 2 School of Optoelectronics, University of Chinese Academy of Sciences, Beijing 100049, China

**Keywords:** piezoelectric-actuated nanopositioning stage, flexure amplification mechanism, multi-configuration hinge model, load-induced displacement loss model

## Abstract

Piezoelectric-actuated nanopositioning stages commonly employ flexure amplification mechanisms to enlarge output displacement and are widely used in optical measurement, micro and nano manufacturing, and other precision engineering fields. However, existing methods still have limitations in computational efficiency, hinge modeling accuracy, and displacement prediction under external loading. To address these limitations, this paper proposes a generalized compliant-chain modeling method in which flexure hinges are treated as compliant units connected by rigid elements, improving computational efficiency relative to repeated finite element modeling. A multi-configuration hinge model (MCH model) is established to analyze the effects of hinge configurations and mechanism parameters on key performance indices, thereby improving prediction accuracy and extending the design space. A load-induced displacement loss model (LDL model) is further developed to characterize output displacement loss under external loading and improve the applicability of the method to loaded conditions and integrated systems involving multiple flexure mechanisms. Finite element simulations and experiments are conducted to validate the proposed models. The results show that the prediction errors of the amplification ratio and stiffness are both within 5%, while the designed mechanism achieves a high amplification ratio of 17.55. These results indicate that the proposed method provides competitive prediction accuracy and displacement amplification performance among similar flexure amplification mechanisms. The proposed method provides an effective modeling and design tool for amplification mechanisms requiring large stroke and high load capacity.

## 1. Introduction

Piezoelectric-actuated nanopositioning stages have been widely used in optical measurement, nano manufacturing, and other precision engineering fields. Typical applications include phase shifting in surface form measurement [1], precision sample stages [2], and optical fiber alignment in confocal measurement systems [3]. Among these applications, phase shifting interferometry (PSI) in white light interferometry requires positioning accuracy and displacement resolution at the nanometer level [4]. Depending on the size and morphology of the measured surface, the stage must also provide a motion stroke ranging from hundreds of micrometers to the millimeter scale, while the use of larger aperture objectives further increases the load capacity requirement. Therefore, the development of precision positioning stages with high accuracy, large stroke, and high load capacity has become a key requirement in precision surface form measurement.

Piezoelectric ceramics (PZT) have become primary actuators in nanopositioning stages because of their high displacement resolution. However, their free stroke is usually limited, and flexure amplification mechanisms are commonly employed to enlarge the output displacement of PZT actuators [5,6,7]. These mechanisms offer compact size and motion without backlash. The design of flexure mechanisms is essentially a multi-objective optimization problem involving coupled dynamics, statics, and kinematics. A design with high performance must balance multiple competing objectives, including displacement amplification, positioning accuracy, structural stiffness, compactness, and load capacity, which are often mutually constrained. Engineering constraints such as stress concentration, fatigue, and the minimum manufacturable feature size must also be considered. These coupled requirements make the design of flexure amplification mechanisms challenging.

Conventional design methods for flexure mechanisms mainly include the finite element method (FEM), topology optimization, and analytical methods [8]. The finite element method provides high accuracy, is easy to implement, and has been extensively developed [9]. However, high-fidelity FEM is computationally expensive and is therefore not well suited to large scale optimization; it is more appropriate for final-stage validation. Topology optimization achieves structural design by redistributing material [10]. However, it may generate slender and weak hinges that are difficult to manufacture, and it can also suffer from numerous numerical instabilities and singular solutions [11]. As a result, topology optimization is more suitable for preliminary topology determination or relatively simple tasks such as lightweight design. Analytical methods simplify flexure mechanisms as compliant chains composed of alternating rigid and compliant bodies, and derive the force displacement relationships using theoretical mechanics [12]. These methods have clear physical principles, low computational cost, and are suitable for rapid design and optimization. However, several limitations remain. First, conventional analytical methods often simplify the compliant body as a single cantilever beam, which reduces prediction accuracy and makes it difficult to account for fillets introduced by practical manufacturing. Second, the influence of hinge geometry on the amplification ratio, stiffness, and stress distribution is often neglected, which restricts the design space and makes it difficult to obtain an optimal solution. In addition, most existing studies focus on isolated amplification mechanisms, whereas in practical applications, fixed loads and the equivalent elastic loads introduced by other parallel flexure mechanisms, such as parallel leaf spring guiding mechanisms, can cause output displacement loss. To the best of our knowledge, these issues have received limited systematic attention.

To address these issues, this study establishes a multi-configuration hinge model (MCH model) to systematically reveal the influence of different hinge configurations on the performance of amplification mechanisms. Specifically, the compliance matrices of leaf, filleted leaf, and V-shaped flexure hinges are derived, and the stress concentration effects in the filleted leaf and V-shaped hinges are analyzed based on notch theory. The results show that the V-shaped hinge exhibits a significant stiffness advantage, thereby providing a broader design space for amplification mechanisms. A load-displacement-loss model (LDL model) is further developed to quantify the displacement loss caused by preload, external load, and equivalent elastic loads imposed by connected mechanisms. By integrating these models with a compliant chain model composed of alternating rigid and compliant bodies, a generalized model for flexure amplification mechanisms is established. The proposed model is validated through finite element analysis (FEA) and experiments. The results demonstrate that the proposed model provides satisfactory prediction accuracy for the key performance indices and load-induced displacement loss. Based on the proposed model, sensitive parameters and optimization rules are summarized, and a bridge-type amplification mechanism with high amplification ratio and favorable dynamic performance is designed. These results demonstrate that the proposed modeling and design method can be effectively applied to the design of flexure amplification mechanisms operating under large-stroke and high-load conditions.

## 2. Analytical Modeling

### 2.1. Common Configurations of Displacement Amplification Mechanisms

**Figure 1:**
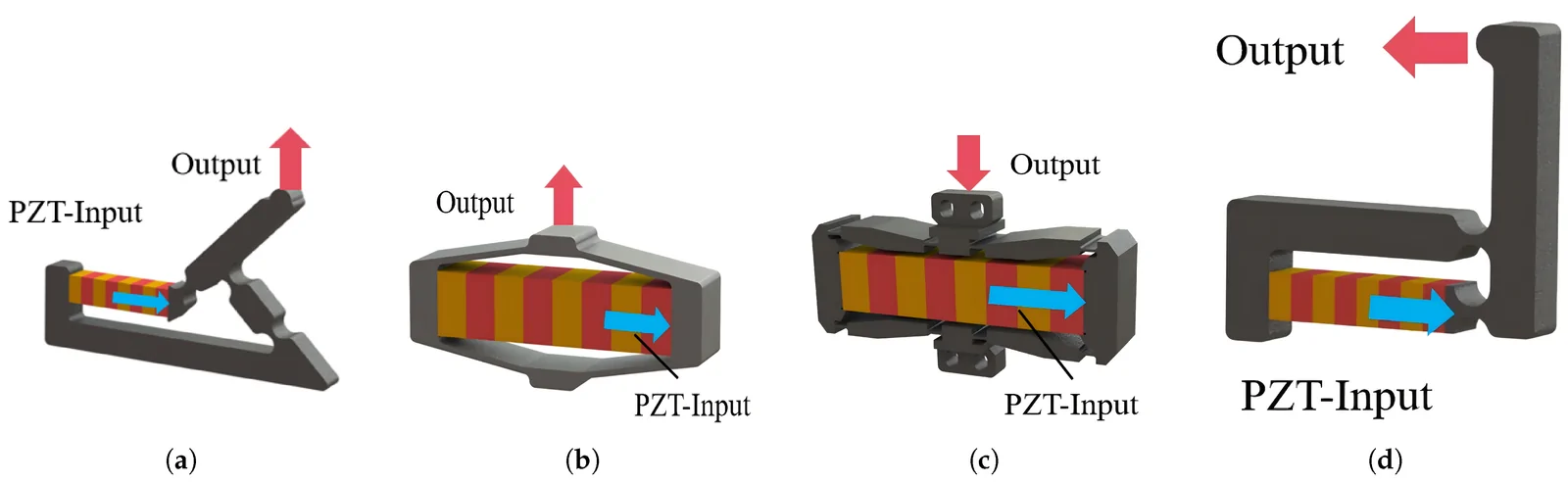
Configurations of displacement amplification mechanisms: (**a**) S–R mechanism. (**b**) Rhombic mechanism. (**c**) Bridge-type mechanism. (**d**) Lever mechanism.

Common displacement amplification mechanisms include Scott–Russell (S–R) mechanisms [13], rhombic mechanisms [14], bridge-type mechanisms [15] and lever mechanisms [16]. The S–R mechanism (Figure 1a) can achieve small parasitic output motion, but it has a relatively complex structure and requires considerable installation space. The rhombic mechanism and the bridge-type mechanism (Figure 1b,c) amplify the output displacement of the PZT actuator through geometric amplification. Owing to their structural symmetry, small parasitic motion, considerable amplification ratio, and compact size, these two mechanisms have been widely used in piezoelectric positioning systems. The lever amplification mechanism (Figure 1d) enlarges displacement based on the lever principle and features a simple structure and straightforward design but has a larger footprint and more pronounced parasitic motion. Although the proposed modeling and design method is applicable to these amplification configurations, the bridge-type mechanism is selected as a representative example in the following analysis because it is widely used [17].

### 2.2. Geometric Model of the Bridge-Type Amplification Mechanism

**Figure 2:**
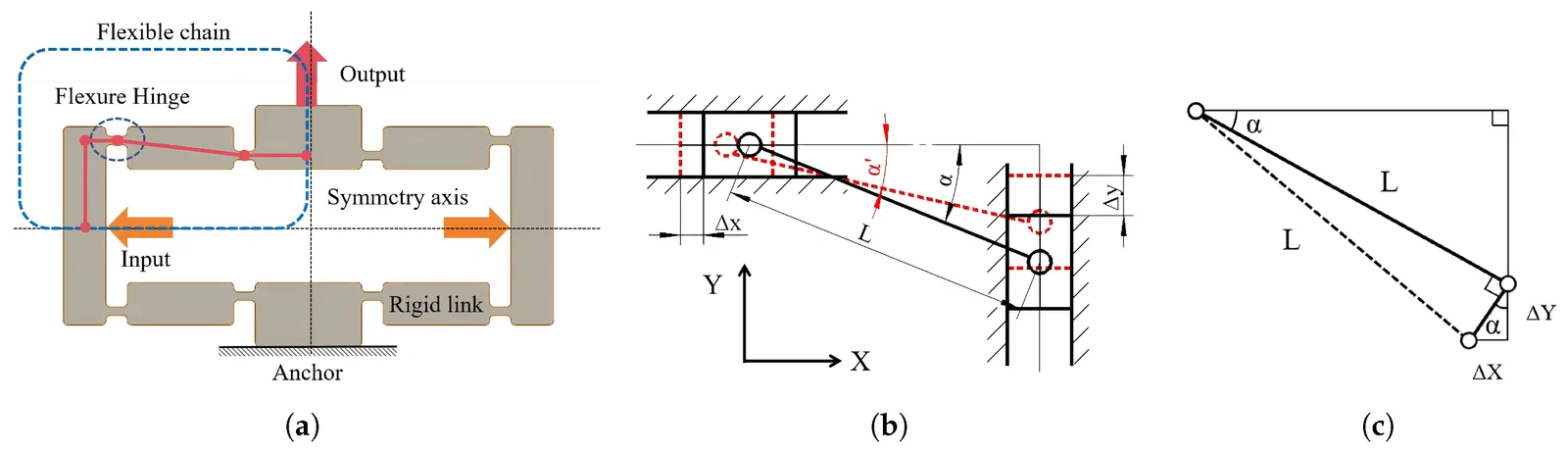
Schematic of the bridge-type mechanism: (**a**) Overall configuration. (**b**) Geometric model of the bridge arm. (**c**) Approximation for the case of Δx≪L.

The bridge-type mechanism has a symmetric configuration (Figure 2a). Owing to this symmetry, one quarter of the mechanism can be defined as a bridge arm. Each bridge arm can be regarded as a link (Figure 2b) whose two ends are connected to sliders moving along the *X* and *Y* directions. The link and the sliders are connected through flexure hinges. Here, *L* and α denote the length and inclination angle of the inclined link.

The geometric relationship between the input displacement Δx and the output displacement Δy of the bridge arm can then be expressed as

(1) $${\left(L\cos\alpha +\Delta x\right)}^{2}+{\left(L\sin\alpha -\Delta y\right)}^{2}=L^{2}.$$

Accordingly, the displacement amplification ratio of the mechanism is given by

(2) $$A=\frac{\Delta y}{\Delta x}=\frac{L\sin\alpha -L\sqrt{1-{\left(\cos\alpha +\frac{\Delta x}{L}\right)}^{2}}}{\Delta x}.$$

When Δx≪L, defining ε=Δx/L (ε≪1), the square root term can be approximated using the first-order Taylor expansion:

(3) $$\sqrt{1-{\left(\cos\alpha +\varepsilon \right)}^{2}}\approx \sin\alpha -\frac{\varepsilon \cos\alpha }{\sin\alpha }.$$

Therefore, the amplification ratio becomes

(4) $$A\approx \frac{\cos\alpha }{\sin\alpha }=\cot\alpha .$$

As illustrated in Figure 2c, when Δx≪L, the connecting rod can be regarded as rotating about its endpoint, thus the same conclusion can be drawn, indicating that the geometric amplification is deter by the inclination angle α under quasi-static conditions. This model provides an initial estimate of the amplification ratio but neglects flexure deformation and external loading effects.

### 2.3. Compliant-Chain Static Modeling

The deformation of flexure amplification mechanisms is mainly concentrated in the flexure hinges. Under the assumptions of small rotation, the deformation of flexure hinges can usually be described using a linear deformation model. Common modeling approaches for flexure mechanisms include the energy method [18], screw theory [19], the compliance matrix method [20], and the pseudo rigid body method [21].

The energy method is commonly used for dynamic modeling. Screw theory uses twists and wrenches to describe the motion and constraint characteristics of flexure mechanisms. It is suitable for systematic kinematic and elastokinematic analysis, but its formulation is relatively abstract and less intuitive for rapid parameter design. The pseudo rigid body method is mainly used to describe the nonlinear behavior of compliant structures undergoing large deformation. Its formulation is relatively complex and is usually more suitable for slender beam flexures or large-displacement compliant mechanisms. The compliance matrix method has a compact formulation and clear physical interpretation, and it can effectively characterize the force-displacement relationship of flexure hinges. This study therefore adopts the compliance matrix method to describe the local mechanical behavior of flexure hinges. A compliant chain consisting of rigid links, flexure hinges, and boundary constraints is then analyzed to establish a generalized static model for flexure amplification mechanisms.

#### 2.3.1. Compliance Model of Flexure Hinges

**Figure 3:**
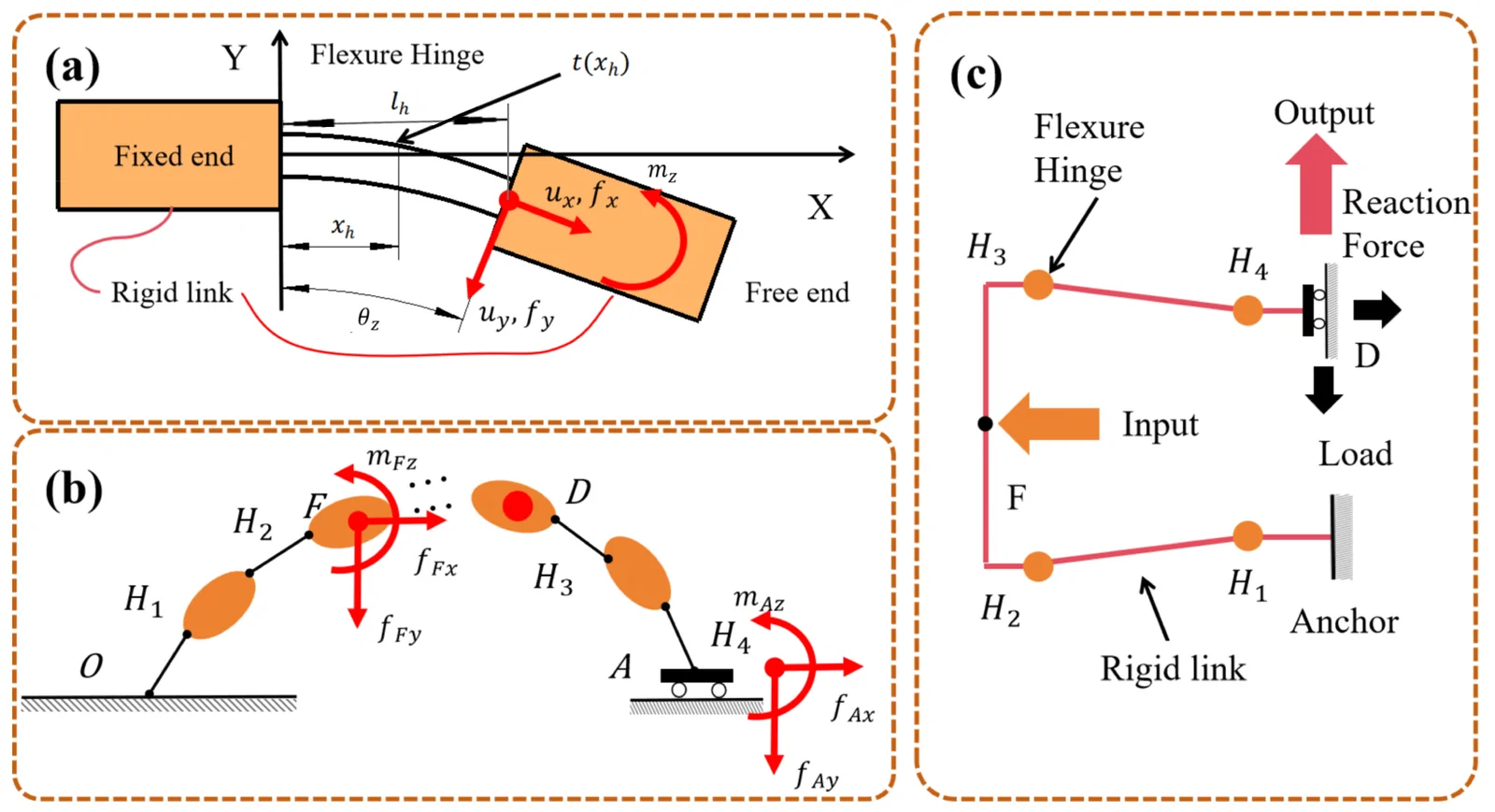
Mechanical models of the amplification mechanism: (**a**) Flexure hinge model. (**b**) Compliant chain model. (**c**) Compliant chain model of the bridge-type mechanism.

The flexure hinge is the basic element that produces elastic deformation in a flexure amplification mechanism. The flexure hinge (Figure 3a) can be regarded as a flexible beam element with one end taken as the fixed reference and the other end allowed to deform under an external load, while both ends are connected to rigid links.

A local coordinate system is established with the fixed end of the flexure hinge as the origin. For planar amplification mechanisms, the end deformation of the flexure hinge includes translations along the *X* and *Y* directions and rotation about the *Z* axis. Under the linear assumption, the relationship between the end displacement and the end load of the flexure hinge can be expressed as

(5) u=Cf,

where the displacement vector, load vector, and compliance matrix are defined as

(6) $$u={\left[\begin{array}{ccc} u_{x} & u_{y} & \theta_{z} \end{array}\right]}^{T},$$

(7) $$f={\left[\begin{array}{ccc} f_{x} & f_{y} & m_{z} \end{array}\right]}^{T},$$

(8) $$C=\left[\begin{array}{ccc} C_{11} & 0 & 0\\ 0 & C_{22} & -C_{23}\\ 0 & -C_{23} & C_{33} \end{array}\right].$$

Here, u, f, and C denote the end displacement vector, load vector, and compliance matrix of the flexure hinge, respectively. The positive directions of the displacement and load components are defined as shown in Figure 3a. The parameters of the hinge shown in the figure include the hinge width *w*, Young’s modulus *E*, local thickness t(x), cross-sectional area A(x), and second moment of area $I_{z}\left(x\right)$, where *x* ranges from 0 to $l_{h}$. The expressions of the compliance coefficients $C_{ij}$ are given in Appendix A.1.

#### 2.3.2. Force–Displacement Formulation of the Compliant Chain

Since the flexure hinge models are established in different local coordinate systems, their local load vectors and displacement vectors need to be transformed into the global coordinate system. Based on the D–H convention for coordinate assignment [22], coordinate transformations are performed using the matrices R and T in homogeneous coordinate form. The transformation can be written as

(9) $$\left\{\begin{array}{c} u=RT^{T}u_{h},\\ f=RTf_{h}. \end{array}\right.$$

In the compliant-chain model, the links are treated as rigid bodies, whereas the hinges are treated as compliant bodies. The alternating connection of rigid links and flexure hinges forms the serial compliant chain (Figure 3b). For the bridge-type amplification mechanism, the structure can be divided along its centerline into two symmetric compliant chains. Owing to this symmetry, only one side of the mechanism is analyzed (Figure 3c). The compliant chain has a fixed starting end, an intermediate rigid body driven by the PZT actuator to generate the input displacement, and a terminal end that produces output displacement along a prescribed direction. During displacement transmission, the deformation generated by the preceding flexure hinge is transferred and amplified through the intermediate rigid link, and then acts as the input displacement of the next flexure hinge. The final output displacement is obtained by accumulating the displacement contributions of all hinges.

Assume that the compliant chain contains *n* hinges and is subjected to *m* external loads. For a given external load *f*, let *D* denote the point where the displacement is to be evaluated, *F* denote the load application point, and $H_{i}$ denote the end of the *i*th flexure hinge from the fixed end. The displacement at point *D* induced by *f* can then be expressed as

(10) $$u_{D}^{f}=C_{H_{1}}f_{F}+\cdots +C_{H_{i}}f_{F}+\cdots +C_{H_{n}}f_{F}=\left(\sum_{i=1}^{n}C_{H_{i}}\right)f_{F}=C_{f}f_{F}.$$

$C_{H_{i}}$ denotes the equivalent compliance contribution of the *i*th flexure hinge to the displacement at point *D* in the global coordinate system. Its calculation involves the following three steps:

1. The external load *f* is transformed into the local coordinate system of hinge $H_{i}$, and the local displacement $u_{i}$ is calculated using Equation (5).
2. The local displacement $u_{i}$ is transformed into the global coordinate system. This transformation can be represented using $T_{FH_{i}}$, $R_{FH_{i}}$, and their transposes.
3. The displacement contribution is translated to point *D*.

$C_{H_{i}}$ can be calculated as

(11) $$C_{H_{i}}=T_{DF}T_{FH_{i}}R_{H_{i}}^{T}C_{i}R_{H_{i}}T_{FH_{i}}^{T}.$$

When multiple external loads are applied, the total output displacement can be obtained by superimposing the displacement contributions induced by all loads:

(12) $$u_{D}=C_{F_{1}}f_{1}+\cdots +C_{F_{j}}f_{j}+\cdots +C_{F_{m}}f_{m}=\sum_{j=1}^{m}\left(C_{f_{j}}f_{j}\right).$$

Due to the symmetry of the bridge-type amplification mechanism, the terminal point *A* of the transmission chain is constrained such that its displacement in the *Y* direction and rotation about the *Z* axis are restricted (Figure 3c). The reaction load and the external input load *f* jointly impose the following displacement constraint:

(13) $$u_{A}={\left[\begin{array}{ccc} u_{Ax} & 0 & 0 \end{array}\right]}^{T}.$$

Substituting Equation (12) into the above constraint gives

(14) $$\left\{\begin{array}{c} f_{Ax}=0,\\ f_{Ay}=\frac{-C_{H}^{f(2)}f_{F}\;C_{H,33}^{A}+C_{H}^{f(3)}f_{F}\;C_{H,23}^{A}}{C_{H,22}^{A}C_{H,33}^{A}-C_{H,23}^{A}C_{H,32}^{A}},\\ m_{Az}=\frac{-C_{H,22}^{A}C_{H}^{f(3)}f_{F}+C_{H,32}^{A}C_{H}^{f(2)}f_{F}}{C_{H,22}^{A}C_{H,33}^{A}-C_{H,23}^{A}C_{H,32}^{A}}. \end{array}\right.$$

After the force–displacement relationship of the amplification mechanism is obtained, two key design indices can be calculated. The displacement amplification ratio is defined as

(15) $$A=\frac{u_{D}}{u_{F}},$$

and the output stiffness is defined as

(16) $$K_{\mathrm{out}}=\frac{f/A}{u_{D}},$$

where f/A represents the equivalent output load estimated from the input load and the amplification ratio.

Although this compliant-chain formulation provides a general static model, the conventional single-hinge representation limits the modeling accuracy and the design space. MCH model is further developed in the next section.

### 2.4. Multi-Configuration Hinge Model Based on the Compliance Matrix Method

**Figure 4:**
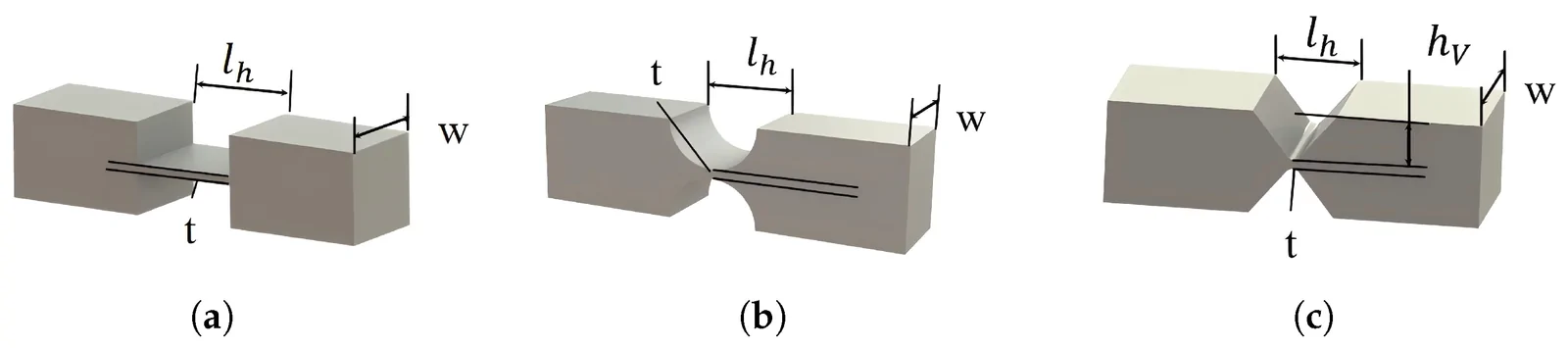
Hinge configurations and design parameters: (**a**) Leaf hinge. (**b**) Filleted leaf hinge. (**c**) V-shaped hinge.

Depending on the thickness profile t(x), flexure hinges can have different geometric forms. Three representative flexure hinge configurations are considered in this study: leaf, filleted leaf, and V-shaped hinges (Figure 4a–c). Establishing mechanical models for different hinge configurations helps improve prediction accuracy and extend the design space of flexure amplification mechanisms.

The common design parameters of these hinges include the length *l*, thickness *t*, and width *w*. For the filleted leaf hinge, the fillet radius *R* is also introduced. When R=0, the filleted leaf hinge reduces to a leaf hinge; when R=l/2, it becomes a circular-arc hinge. Since hinges are commonly fabricated by wire cutting, a leaf hinge inevitably contains a fillet radius no smaller than the wire radius. The filleted leaf hinge is therefore more consistent with practical manufacturing conditions and can improve prediction accuracy. The V-shaped hinge has an additional design parameter, namely the cutting depth $h_{v}$. Different hinge configurations involve different trade-offs among fatigue performance, displacement amplification, and structural stiffness, and their derived compliance matrices are given in Appendix A.1.

In hinge design, the maximum stress calculation is an important basis for strength and fatigue life verification. Under the modeling assumptions established in the previous section, shear stress and torsional stress can be neglected. The maximum normal stress is obtained by superimposing the axial normal stress and the maximum bending normal stress at the edge:

(17) $$\sigma_{\max}=\sigma_{a}+\sigma_{b,\max}.$$

Different hinge configurations have different axial and bending stress concentration factors, denoted by $K_{a}$ and $K_{b}$:

(18) $$\begin{array}{cc} \sigma_{a} & =K_{a}\frac{F_{x}}{wt},\\ \sigma_{b,\max} & =K_{b}\frac{6(F_{y}l+M_{z})}{wt^{2}}. \end{array}$$

The filleted leaf and V-shaped hinges are treated as notches in a tensile plate. The stress concentration factor of the V-shaped flexure hinge is calculated using Williams’ notch stress field theory [23], and that of the filleted leaf flexure hinge is obtained using an extension of Inglis’ elliptical notch theory [24] (see Appendix A.2 for the detailed derivations).

### 2.5. Dynamic Model of the Amplification Mechanism

Starting from the static model established in the previous section, a dynamic model of the amplification mechanism is developed to estimate the natural frequency of the system. Since the output motion of the amplification mechanism mainly occurs along a prescribed direction, the mechanism can be regarded as a system with a single degree of freedom. Based on Lagrange’s method, the displacements, deformations, and input force of the mechanism are equivalently transformed to the output displacement direction. The equivalent output displacement is taken as the generalized coordinate *q*, and the generalized force is denoted as $Q=F_{\mathrm{in}}/A$.

The mass of the mechanism itself is assumed to be negligible compared with the load mass, and the output stiffness obtained from Equation (16) is used to describe the elastic potential energy of the system. The kinetic energy and potential energy can therefore be expressed as

(19) $$\begin{array}{cc} T & =\frac{1}{2}M_{\mathrm{load}}{\dot{q}}^{2},\\ V & =\frac{1}{2}K_{\mathrm{out}}q^{2}. \end{array}$$

The Lagrange equation of the system is given by

(20) $$\begin{array}{cc} \frac{d}{dt}\left(\frac{\partial L}{\partial \dot{q}}\right)-\frac{\partial L}{\partial q} & =Q,\\ L & =T-V. \end{array}$$

Substituting Equation (19) and $Q=F_{\mathrm{in}}/A$ into Equation (20) gives the equation of motion:

(21) $$M_{\mathrm{load}}\ddot{q}+K_{\mathrm{out}}q=\frac{F_{\mathrm{in}}}{A}.$$

The natural frequency of the system is then obtained as

(22) $$f_{d}=\frac{1}{2\pi }\sqrt{\frac{K_{\mathrm{out}}}{M_{\mathrm{load}}}}.$$

## 3. Finite Element Validation

Finite element simulations are performed using ANSYS Workbench 2023 R1 to validate the accuracy of the above models. A linear elastic material is used, with a Young’s modulus of 100 GPa and a Poisson’s ratio of 0.3. Tetrahedral elements are adopted to accommodate the variable cross sections of the flexure hinges, and local mesh refinement is applied to the hinge regions.

### 3.1. Static Validation

**Figure 5:**
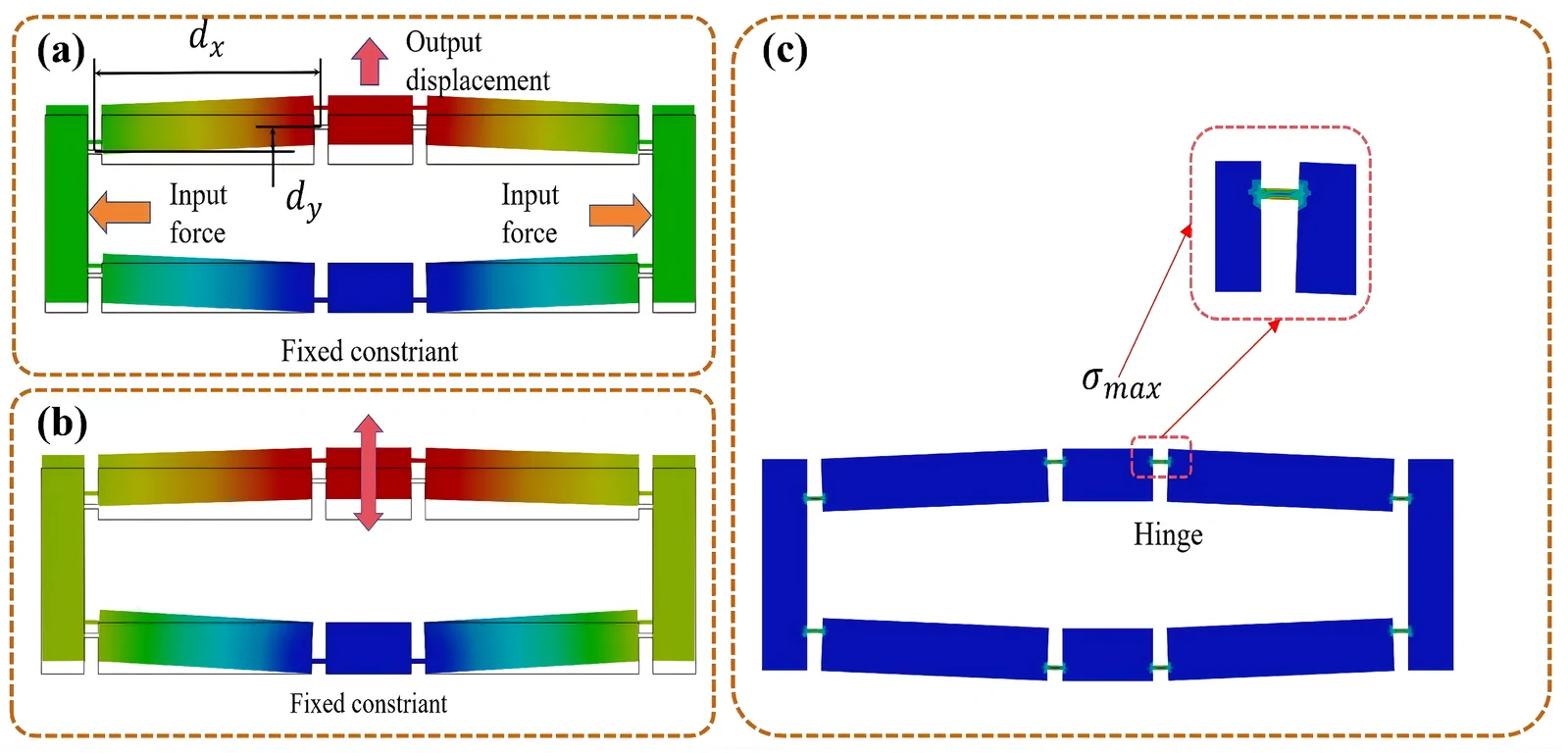
Finite element validation: (**a**) Static model validation. (**b**) von Mises stress. (**c**) Modal simulation validation.

In the static simulation, the fixed end is constrained, and an input force of 50 N is applied at the input end (Figure 5a). The displacement amplification ratio, output stiffness, and maximum von Mises stress (Figure 5b) are calculated from the simulation results.

The design parameters of the bridge-type mechanism are defined according to Figure 4 and listed in Table 1. The transverse and longitudinal distances between the hinges at the two ends of the bridge arm are defined in Figure 5a. By varying $d_{y}$, *l*, *t*, and the hinge configuration, the calculation results of each performance index are obtained and compared with the theoretical model to evaluate the prediction errors.

**Figure 6:**
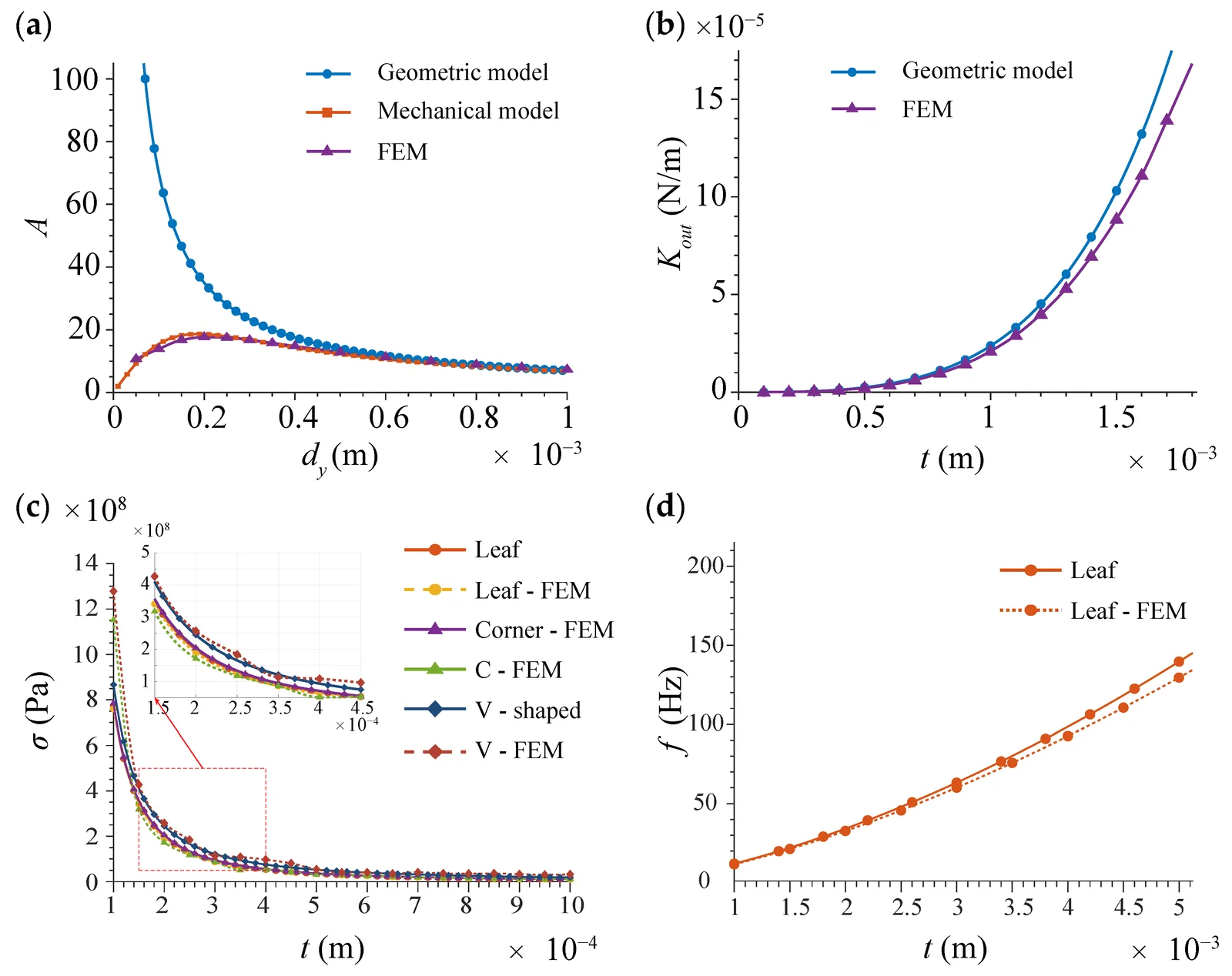
Comparison between theoretical models and finite element results: (**a**) Amplification ratio under different longitudinal distances. (**b**) Stiffness under different hinge thicknesses. (**c**) Stress under different hinge configurations and thicknesses. (**d**) The natural frequency under different hinge thicknesses.

The simulation results reveal the variation trend of the model accuracy. Figure 6a shows that the accuracy of the geometric model decreases significantly when the amplification ratio is large, whereas the mechanical model maintains relatively high accuracy. A limit of the amplification ratio is also observed when the hinge thickness is small. Figure 6b shows that when the hinge thickness exceeds 1 mm, the shear effect in deformation becomes significant, and the cantilever-beam assumption is no longer fully valid, leading to increased stiffness prediction error. Figure 6c shows that the stresses differ among the three hinge configurations, and the MCH model can predict these differences with good accuracy.

### 3.2. Modal Validation of the Dynamic Model

In the modal simulation, the other settings are kept consistent with those in the static simulation, while the material is changed to 6061 aluminum alloy (Figure 5c). The hinge thickness is varied, and natural frequency is compared between the theoretical model and the finite element model.

The modal results show that the first mode of the amplification mechanism is dominated by reciprocating motion of the output end with a single degree of freedom, which is consistent with the model assumption. Figure 6d shows that the variation trend of the model accuracy with hinge thickness is consistent with the output stiffness variation trend shown in Figure 6b. This consistency agrees with the derivation of the dynamic model in Equation (22).

As shown in Table 2, the maximum error of the static model is less than 5%, and the maximum error of the dynamic model is less than 8%. The static error mainly originates from the increasing shear effect caused by the increase in hinge thickness [25]. The dynamic error mainly results from the lumped equivalence of mass and stiffness [26].

The computational time of the theoretical model is significantly shorter than that of the finite element method. For the calculations listed in Table 2, the MATLAB R2022b script requires 2.206 s, whereas the parameterized simulations in ANSYS Workbench require 57 min in total. This comparison indicates that the theoretical model has higher computational efficiency and is more suitable for large-scale parameter optimization because it avoids the repeated procedures of geometry modification, mesh generation, and unnecessary computations in regions with negligible deformation.

## 4. Extended Modeling and Design Optimization of the Amplification Mechanism

Based on the theoretical models established and validated, this section further proposes an amplification efficiency index and a load-induced displacement loss model. These two extensions are used to provide stricter constraints for the optimal design and to improve the prediction accuracy of the output displacement. The established models are then used to calculate the design parameters and performance indices of the amplification mechanism. The influence trends of key design parameters and the corresponding optimization principles are summarized.

### 4.1. Amplification Efficiency

According to the finite element results (Figure 6a), the mechanical amplification ratio first increases and then decreases as the longitudinal hinge distance decreases, indicating the existence of an amplification limit. In contrast, the geometric amplification ratio continues to increase. To characterize the deviation between these two ratios, the amplification efficiency η is defined as the ratio of the mechanical amplification ratio to the geometric amplification ratio:

(23) $$\eta =\frac{A}{G},$$

where *A* denotes the mechanical amplification ratio and *G* denotes the geometric amplification ratio.

**Figure 7:**
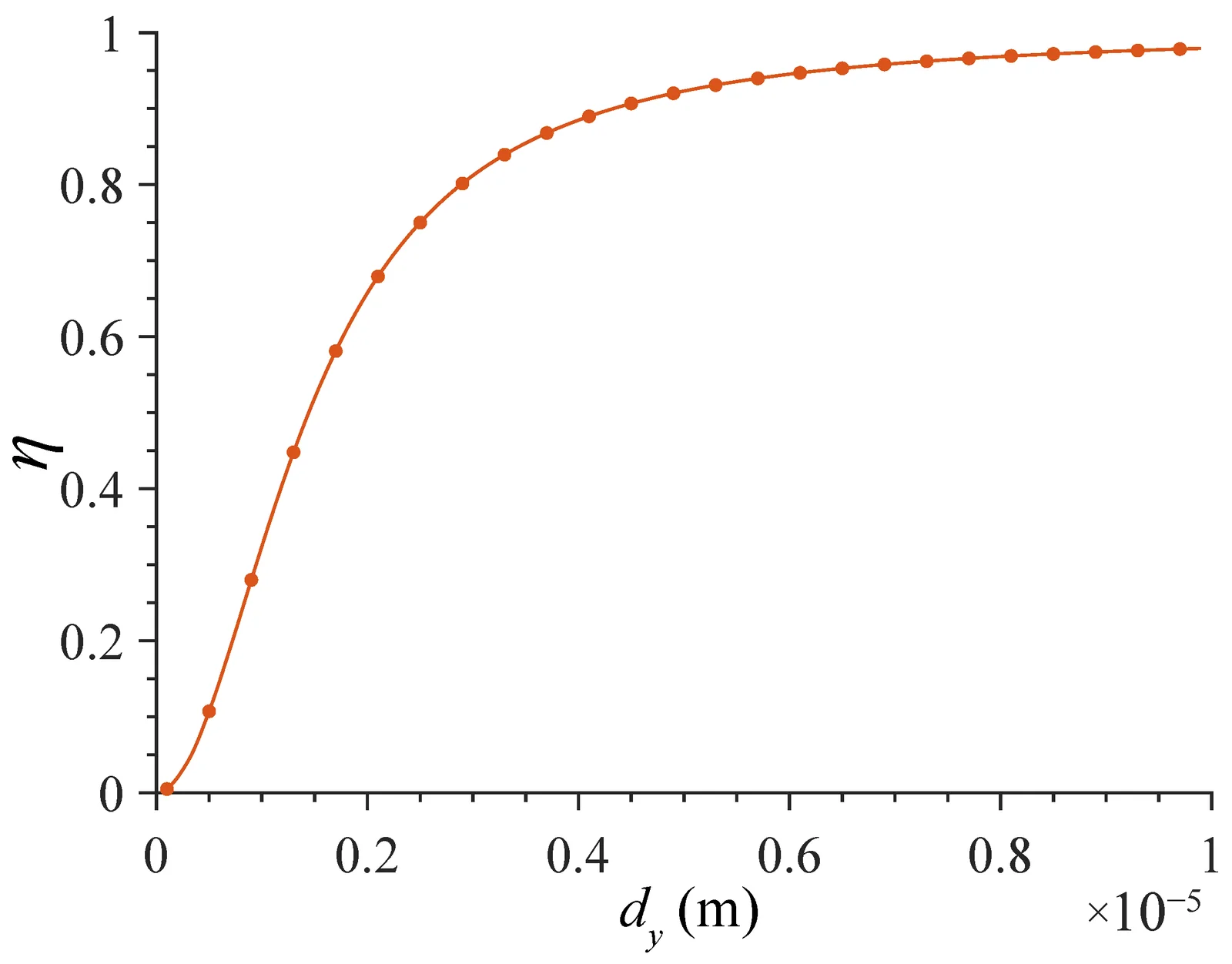
Variation of amplification efficiency with longitudinal hinge distance.

The calculated amplification efficiency shows that, in the smaller longitudinal distance region, the amplification efficiency rapidly decreases from nearly one to nearly zero (Figure 7). In this region, stress stiffening [27] and axis drift [28] become significant, and nonlinear behavior becomes pronounced. The linear modeling assumptions are therefore no longer satisfied. Most of the input energy is used to overcome the structural stiffness and is converted into elastic strain energy, which reduces the energy available for external output. Therefore, the amplification efficiency should be kept as close to one as possible during optimal design.

### 4.2. Load-Induced Displacement Loss Model

The external load applied to the amplification mechanism and the preload applied to prevent tensile loading of the PZT both lead to output displacement loss. This phenomenon is referred to as the load effect. A load-induced displacement loss (LDL) model is established to make the predicted output displacement of the amplification mechanism closer to that under practical operating conditions.

**Figure 8:**
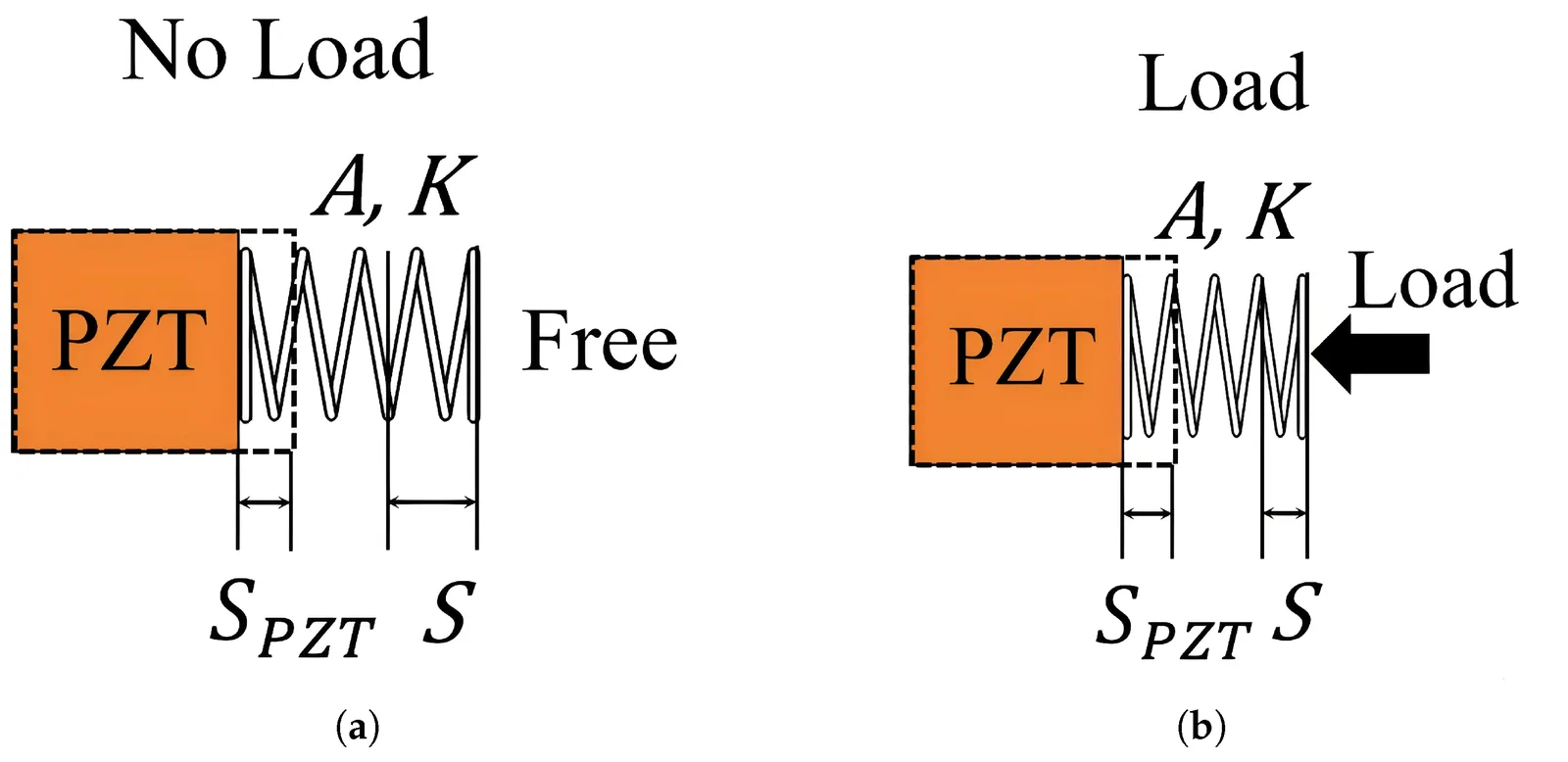
PZT–amplification mechanism model: (**a**) No load. (**b**) Load.

In the no-load model, the PZT is represented by a series model consisting of an ideal displacement source. Its intrinsic stiffness and the amplification mechanism can be regarded as an elastic element with displacement amplification (Figure 8a). The relationship between the output force $F_{\mathrm{PZT}}$ and the actual displacement of the PZT *S* can be written as

(24) $$F_{\mathrm{PZT}}=K_{\mathrm{PZT}}\left(S_{\mathrm{PZT}}-S\right),$$

where $S_{\mathrm{PZT}}$ is the free displacement of the PZT and $K_{\mathrm{PZT}}$ is stiffness of PZT.

Under the no-load condition, the output displacement can be obtained by establishing force equilibrium at the input end of the amplification mechanism:

(25) $$F_{\mathrm{PZT}}=KS,$$

where *K* is the input stiffness of the amplification mechanism. The output displacement is then given by

(26) $$S_{\mathrm{load}}=\frac{AK_{\mathrm{PZT}}}{K+K_{\mathrm{PZT}}}S_{\mathrm{PZT}}=\frac{AS_{\mathrm{PZT}}}{1+\frac{K}{K_{\mathrm{PZT}}}}.$$

In general, the input stiffness of the amplification mechanism is much smaller than the intrinsic stiffness of the PZT. Therefore, the displacement loss caused by the PZT stiffness is limited.

Under a constant load (Figure 8b), the displacement amplification from the input end to the output end reduces the output force by the same ratio according to the work balance. In this case, the PZT output end is subjected to both the deformation reaction force of the amplification mechanism and the load force reflected to the input end. The equilibrium equation and output displacement are

(27) $$\begin{array}{cc} K_{\mathrm{PZT}}\left(S_{\mathrm{PZT}}-S_{\mathrm{load}}\right)\; & =KS+AF_{\mathrm{load}},\\ S_{\mathrm{load}}\; & =\frac{-A^{2}F_{\mathrm{load}}+AK_{\mathrm{PZT}}S_{\mathrm{PZT}}}{K+K_{\mathrm{PZT}}}. \end{array}$$

Using the same analysis procedure, the equilibrium equation and output displacement under an elastic load are

(28) $$\begin{array}{cc} K_{\mathrm{PZT}}\left(S_{\mathrm{PZT}}-S_{\mathrm{load}}\right)\; & =KS+A^{2}K_{\mathrm{load}}S_{\mathrm{load}},\\ S_{\mathrm{load}}\; & =\frac{AK_{\mathrm{PZT}}}{K_{\mathrm{PZT}}+K+A^{2}K_{\mathrm{load}}}S_{\mathrm{PZT}}. \end{array}$$

**Figure 9:**
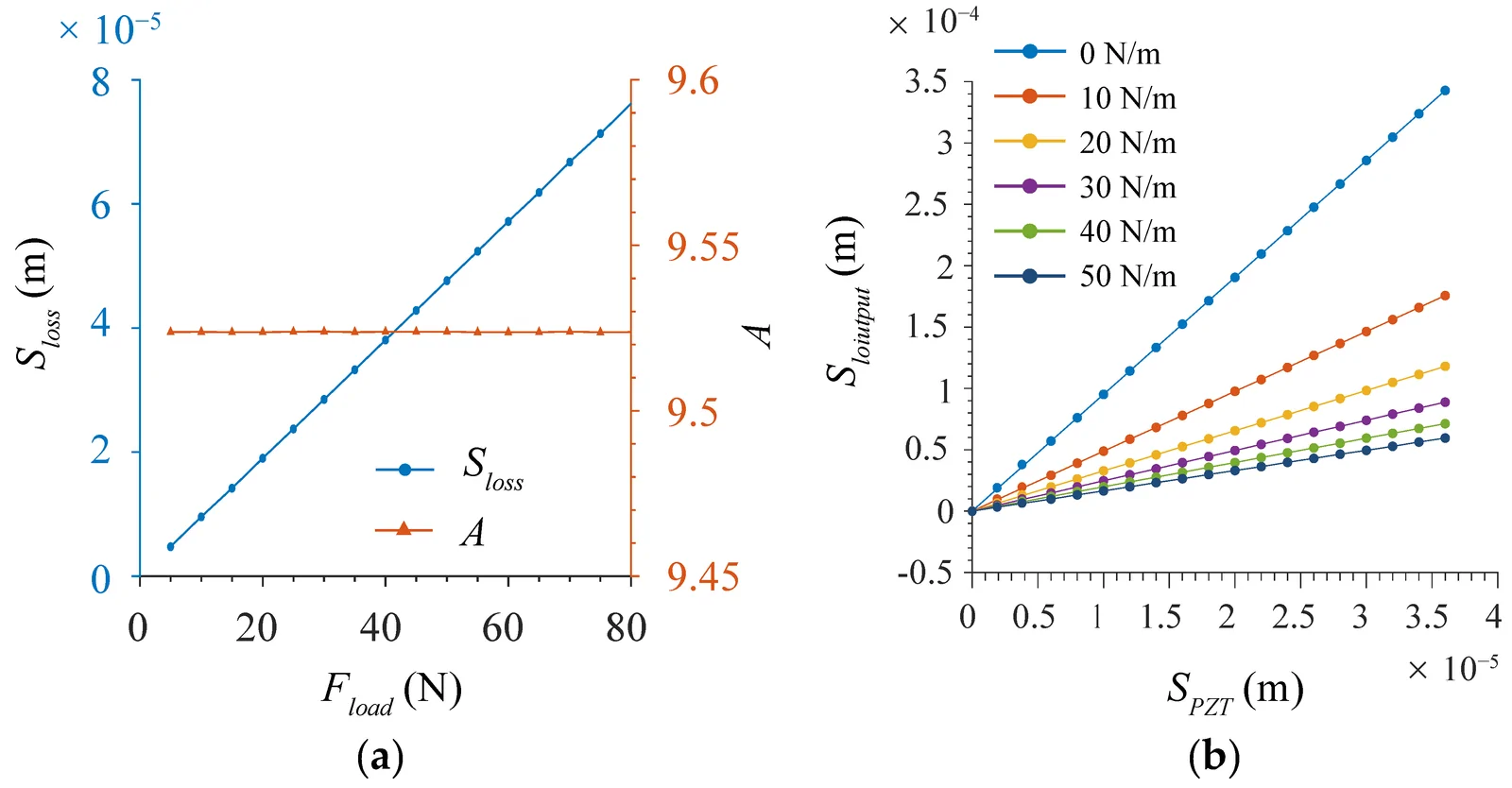
Theoretical calculation results of load-induced displacement loss: (**a**) Displacement loss and amplification ratio under a constant load. (**b**) Output displacement under an elastic load.

The theoretical model established in Section 2.3 is used to validate the LDL model. Under a constant load, the amplification ratio remains unchanged, whereas the output displacement loss increases approximately in proportion to the load (Figure 9a). The PZT preload can also be treated as a constant load. Under an elastic load, the effective output amplification ratio decreases because the load force increases with the output displacement (Figure 9b). The subsequent stages in a multistage amplification mechanism and flexure guiding mechanisms can both be regarded as elastic loads.

According to Equation (27), a larger amplification ratio leads to greater displacement loss and lower load capacity under a constant load. Increasing the output displacement should therefore not rely solely on increasing the amplification ratio. Under an elastic load, Equation (28) shows that $K_{\mathrm{load}}$ is rapidly amplified by $A^{2}$. When $A^{2}K_{\mathrm{load}}$ approaches the intrinsic stiffness of the PZT, the output displacement loss increases significantly. Therefore, the equivalent stiffness of the subsequent flexure mechanisms should be limited such that $A^{2}K_{\mathrm{load}}\ll K_{\mathrm{PZT}}$.

### 4.3. Numerical Parametric Analysis and Design Optimization Method

The design parameters of the amplification mechanism can be divided into three categories. The first category is the hinge configuration. The three hinge configurations have different performance characteristics, and comparing their differences is useful for improving the overall performance of the amplification mechanism. The second category is the hinge dimensional parameters, and the third category is the structural parameters of the mechanism. These two categories constitute the core design variables for optimization. Based on the proposed models, the effects of these parameters on the amplification ratio, natural frequency, load effect, and maximum stress are calculated, and optimization rules for the sensitive parameters are summarized. The other fixed parameters of the amplification mechanism are listed in Table 1.

#### 4.3.1. Hinge Configurations

The mechanism performance of the three hinge configurations are calculated under different hinge lengths and thicknesses. Considering the wire diameter limitation and fracture risk in practical wire cutting, the lower design limit of the hinge thickness and the minimum fillet radius are set to 0.2 mm.

#### 4.3.2. Hinge Dimensional Parameters

**Figure 10:**
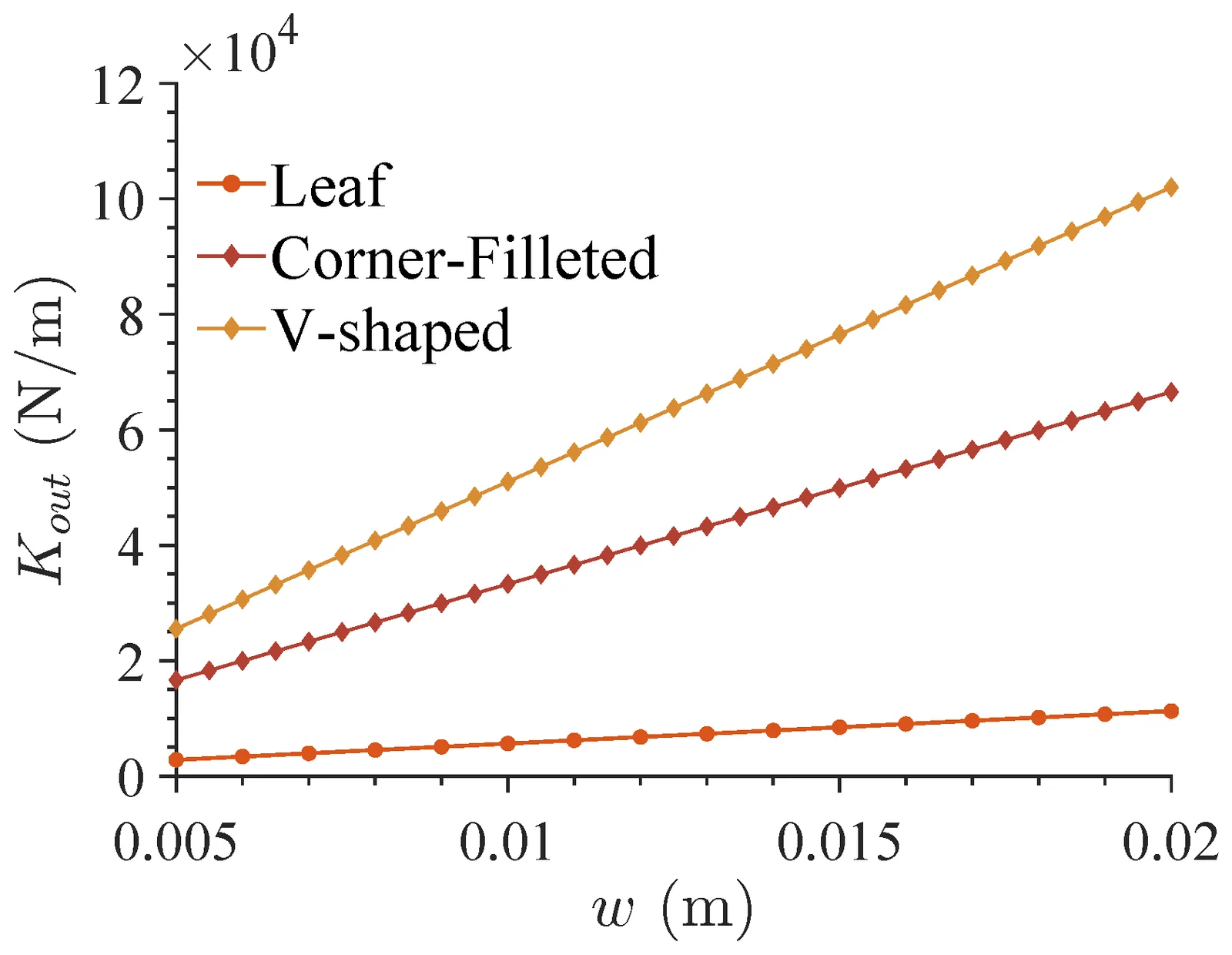
Effect of hinge width on output stiffness.

The effects of hinge length and hinge thickness on the mechanism performance are evaluated in Section 4.3.1. The effects of hinge width, fillet radius of the filleted leaf hinge, and cutting depth of the V-shaped hinge are further calculated. Since the hinge width has an evident effect only on the output stiffness, it is evaluated separately (Figure 10).

#### 4.3.3. Structural Parameters

The structural parameters of the amplification mechanism, namely the transverse and longitudinal hinge distances, can be represented by the angle between the bridge arm and the horizontal direction:

(29) $$\alpha =\arctan\left(\frac{d_{y}}{d_{x}}\right).$$

The effect of the bridge-arm angle on the amplification ratio and output stiffness is calculated within the small-angle range. Since the maximum stress is weakly correlated with the bridge-arm angle, the stress results are not listed.

#### 4.3.4. Design Method Discussion

Based on the calculation results in the preceding three subsections, the sensitivity ranking of parameters can be summarized as follows. Parameters not listed below show no clear correlation with the corresponding performance index.

- Amplification ratio *A*: $\alpha >l>h_{v}$ (V-shaped hinge) >t≈R (filleted leaf hinge);
- Output stiffness $K_{\mathrm{out}}$: t>R (filleted leaf hinge) $\approx w>h_{v}$ (V-shaped hinge) >l>α;
- Maximum stress σ: t>R (filleted leaf hinge) $>h_{v}$ (V-shaped hinge) >l;
- Amplification efficiency η: α>t>l.

**Figure 11:**
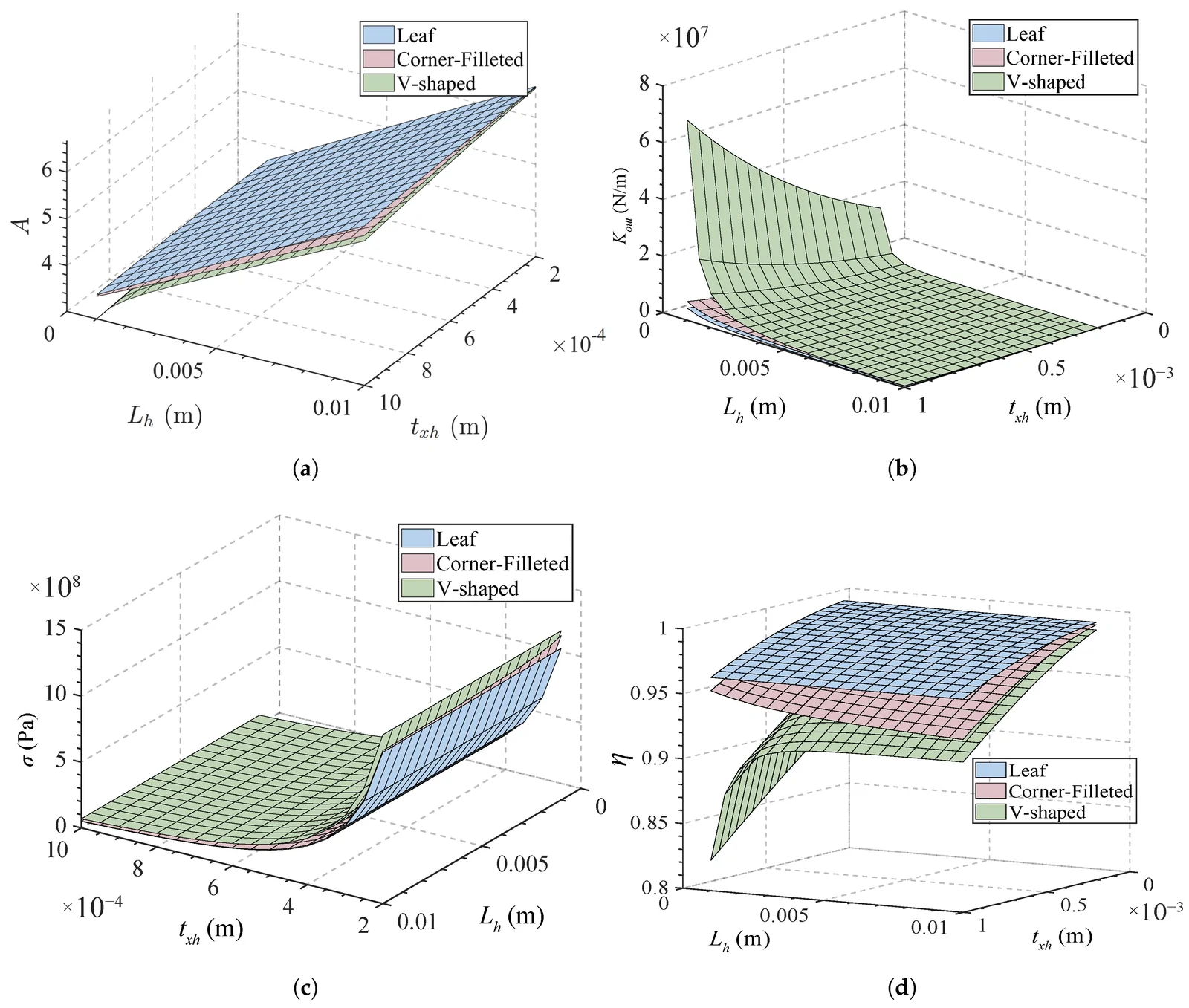
Relationships between hinge parameters and performance indices for the three hinge configurations: (**a**) Amplification ratio. (**b**) Output stiffness. (**c**) Maximum stress. (**d**) Amplification efficiency.

The calculated results show that the filleted leaf hinge and the leaf hinge have similar performance characteristics (Figure 11a–d). For the V-shaped hinge, the output stiffness increases sharply and the amplification efficiency decreases rapidly as the hinge thickness increases and length decreases. Its performance variation range is therefore much larger than those of the other two hinge configurations. However, the maximum stress of the V-shaped hinge is also higher.

**Figure 12:**
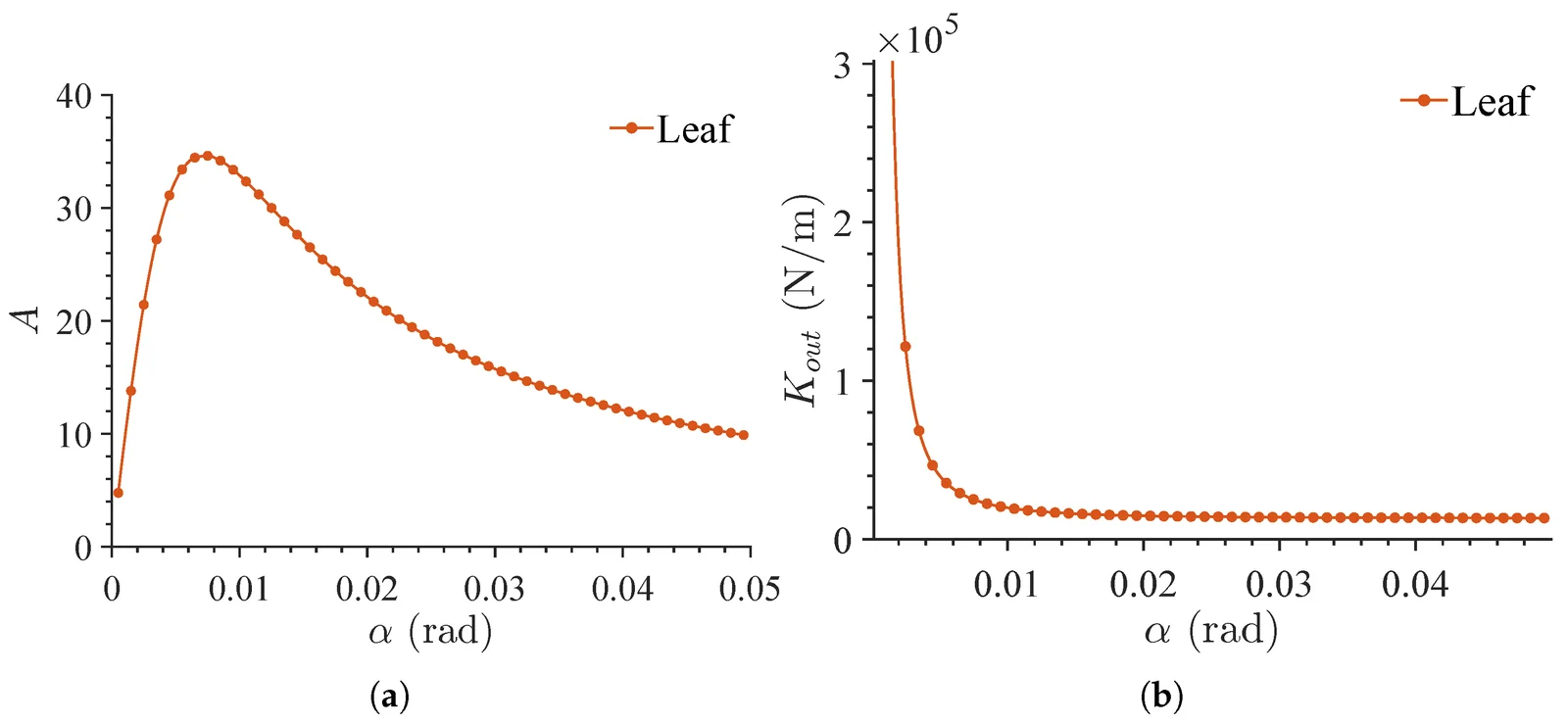
Effects of bridge-arm angle on mechanism performance: (**a**) Bridge-arm angle and amplification ratio. (**b**) Bridge-arm angle and output stiffness.

The bridge-arm angle mainly affects the amplification ratio and amplification efficiency (Figure 11 and Figure 12). When the bridge-arm angle enters the region below the amplification limit, the amplification efficiency decreases sharply and the output stiffness increases rapidly. Therefore, the bridge-arm angle should be selected above the amplification-limit region. The hinge width only affects the output stiffness and has no evident influence on the other performance indices (Figure 10). Thus, increasing the hinge width should be prioritized when only the output stiffness needs to be improved.

**Figure 13:**
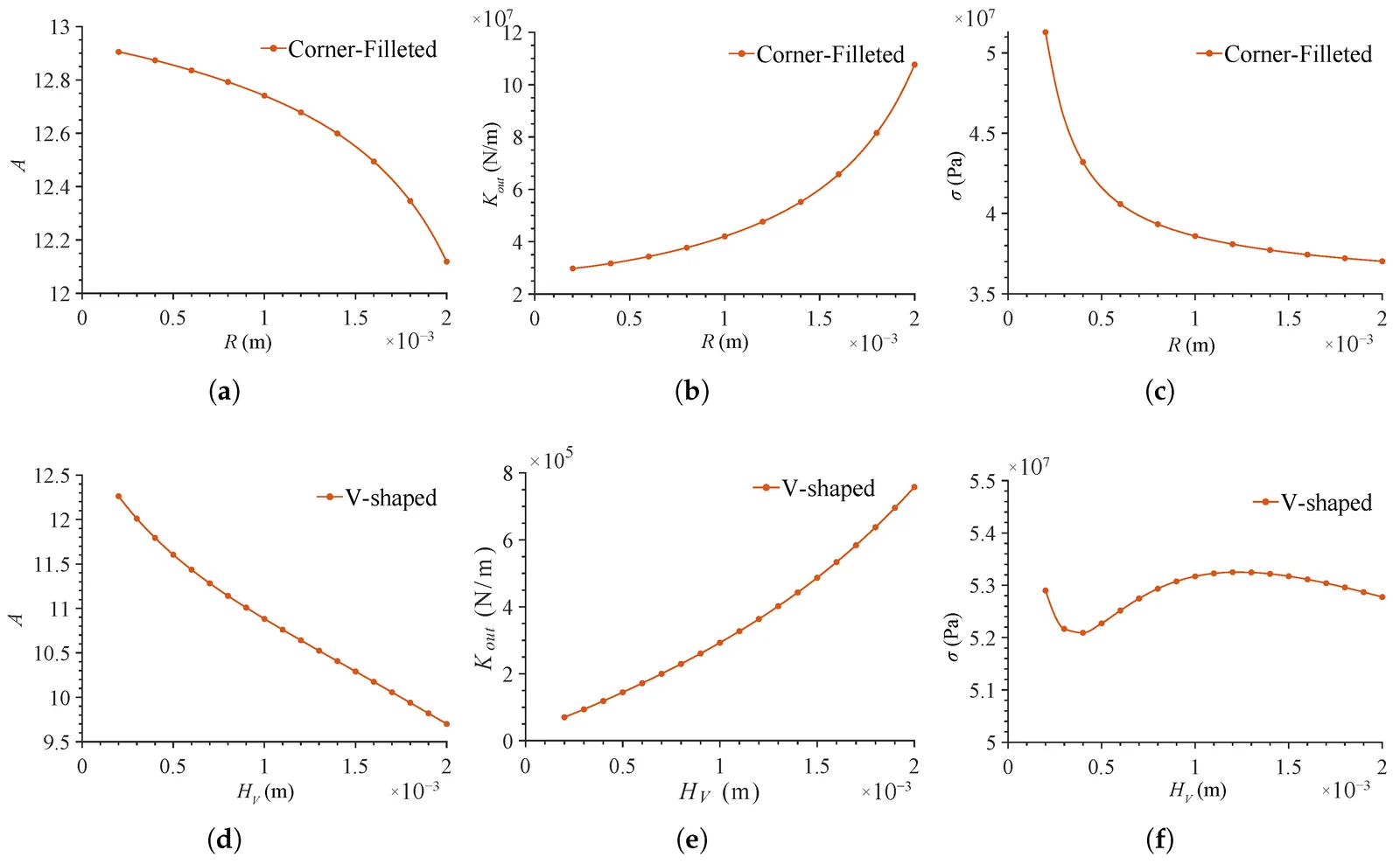
Effects of fillet radius and cutting depth on mechanism performance: (**a**) Fillet radius and amplification ratio. (**b**) Fillet radius and output stiffness. (**c**) Fillet radius and maximum stress. (**d**) Cutting depth and amplification ratio. (**e**) Cutting depth and output stiffness. (**f**) Cutting depth and maximum stress.

The hinge thickness increases the output stiffness and reduces the maximum stress, which is consistent with the general rules of mechanics of materials (Figure 11b,c). The effects of the fillet radius and the cutting depth are similar to that of the hinge thickness because these two parameters change the local equivalent thickness (Figure 13 and Figure 11b,c). They can improve the output stiffness and reduce the maximum stress while keeping the amplification ratio nearly unchanged. This helps improve the dynamic characteristics and reduce displacement loss. Stress concentration at the bottom of the V-shaped notch limits the reduction in maximum stress. Therefore, stricter strength verification is required when V-shaped hinges are used.

Based on the above rules, the design procedure of the amplification mechanism can be summarized as follows:

1. Adjust the bridge-arm angle to preliminarily design the amplification ratio and keep the amplification efficiency within an acceptable range.
2. Use the leaf hinge for the preliminary design of the amplification ratio and output stiffness because of its simple calculation.
3. Replace the leaf hinge with the filleted leaf hinge and improve the design accuracy by considering the minimum manufacturable feature size.
4. Adjust the hinge dimensional parameters to optimize the output stiffness and maximum stress.
5. Determine whether to replace the original hinges with V-shaped hinges to improve the output stiffness. The limitations imposed by the maximum stress and amplification efficiency should be considered.
6. Use the LDL model to calculate the displacement loss under the actual load and complete the overall design optimization.

## 5. Experimental Validation and Discussion

Experiments are conducted to validate the prediction accuracy of the proposed models and preliminarily examine the feasibility of the design method. The parametric analysis in the previous section shows that the hinge thickness and the bridge-arm angle are the most sensitive parameters. Prototypes are fabricated and tested with these two parameters varied. For manufacturing convenience, filleted leaf hinges are selected. The other fixed parameters are listed in Table 3.

### 5.1. Experimental Apparatus

#### 5.1.1. Experimental Environment and Materials

**Figure 14:**
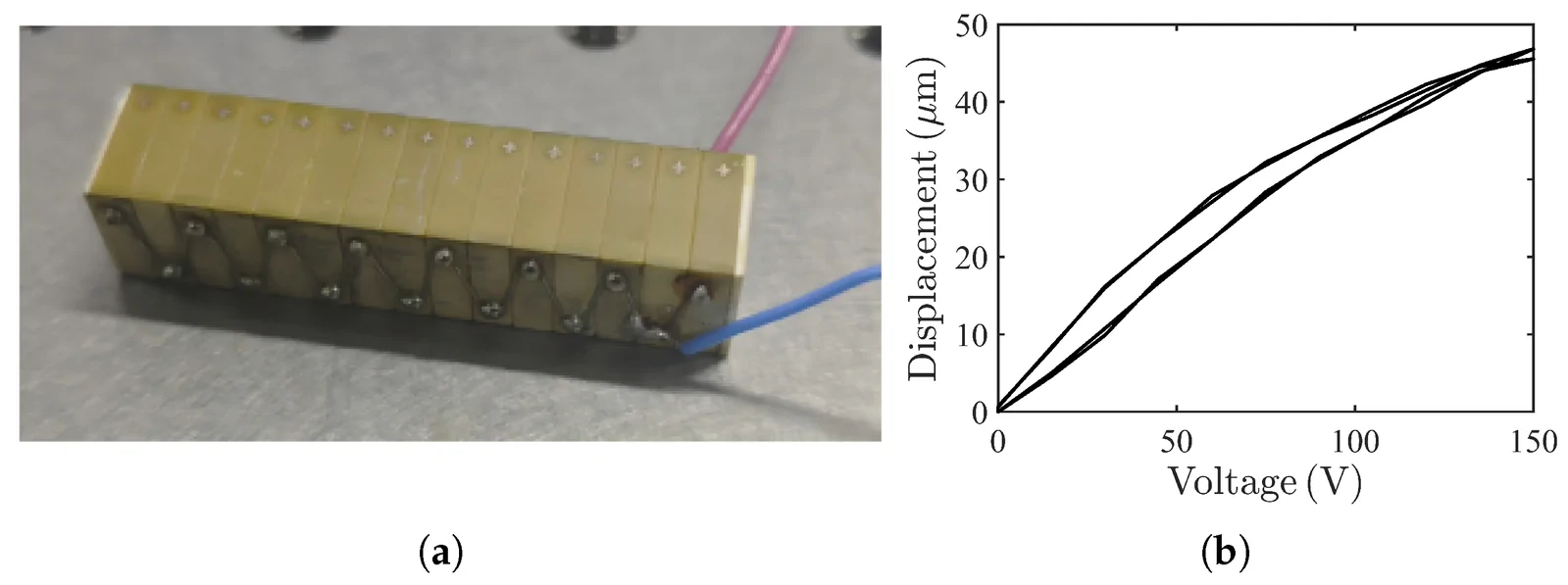
PZT stack: (**a**) Physical photograph. (**b**) Relationship between PZT displacement and voltage.

The PZT used in the experiment is a PZT-5H stack with a nominal size of 10×10×40 mm. Its maximum free displacement is 45.5 μm, the operating voltage range is 0–120 V, and the theoretical maximum output force is 4000 N. The displacement output of the PZT stack is measured using a laser interferometer (Figure 14b).

All experimental setups are fixed on an optical table equipped with pneumatic vibration isolators. The experiments are conducted in a laboratory with controlled temperature (22±2 °C) and humidity (50±5%).

#### 5.1.2. Stiffness Measurement Apparatus

**Figure 15:**
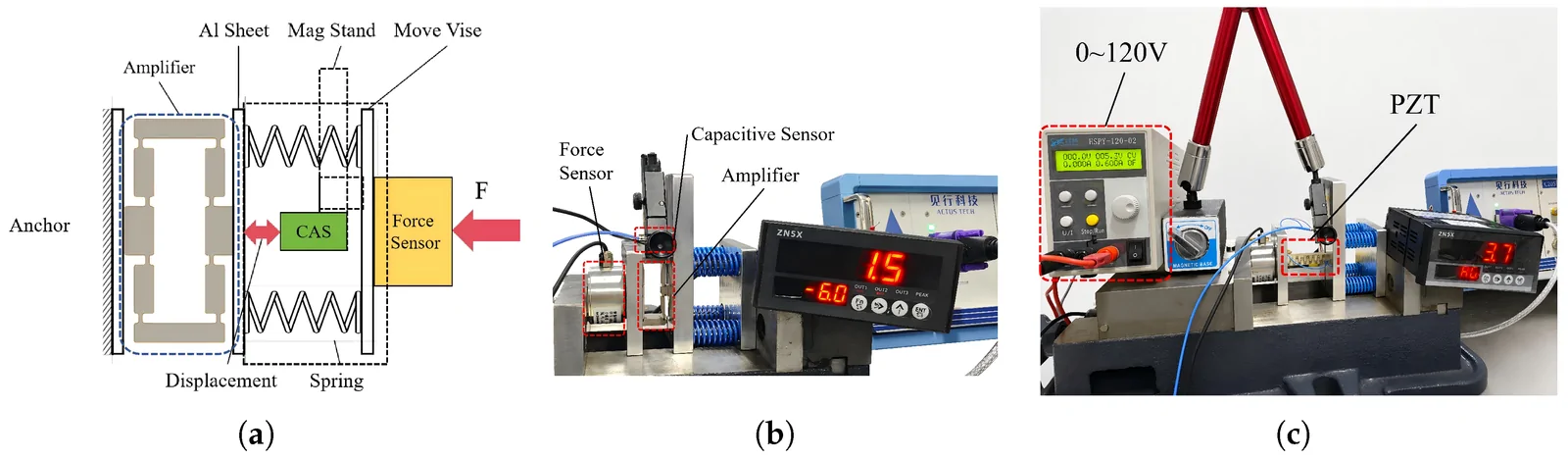
Stiffness measurement apparatus: (**a**) Schematic (CAS is capacitance sensor). (**b**) Amplification-mechanism stiffness test. (**c**) PZT stiffness test.

An apparatus is built to test the force, displacement, and stiffness of the amplification mechanism and the PZT. The measurement principle is to calculate the stiffness from the simultaneously measured displacement and load. A low-stiffness spring is connected in series at the loading end to improve the load resolution (Figure 15a). A load is applied to both ends of the tested object using a vise, while the force is measured using a force sensor (ZNLBM-7, resolution: 0.1 N) and the displacement is measured using a capacitive displacement sensor (C202, resolution: 0.75 nm).

The base of the capacitive displacement sensor moves with the vise, so the measured displacement is equal to the displacement of the tested object. For stiffness measurement of the amplification mechanism, the output end of the mechanism is clamped between the jaws of the vise, and the load is applied gradually. For stiffness measurement of the PZT, a voltage of 120 V is applied using an HSPY120-02 power supply, and a similar measurement procedure is then repeated.

#### 5.1.3. Amplification Ratio and Displacement Loss Measurement

**Figure 16:**
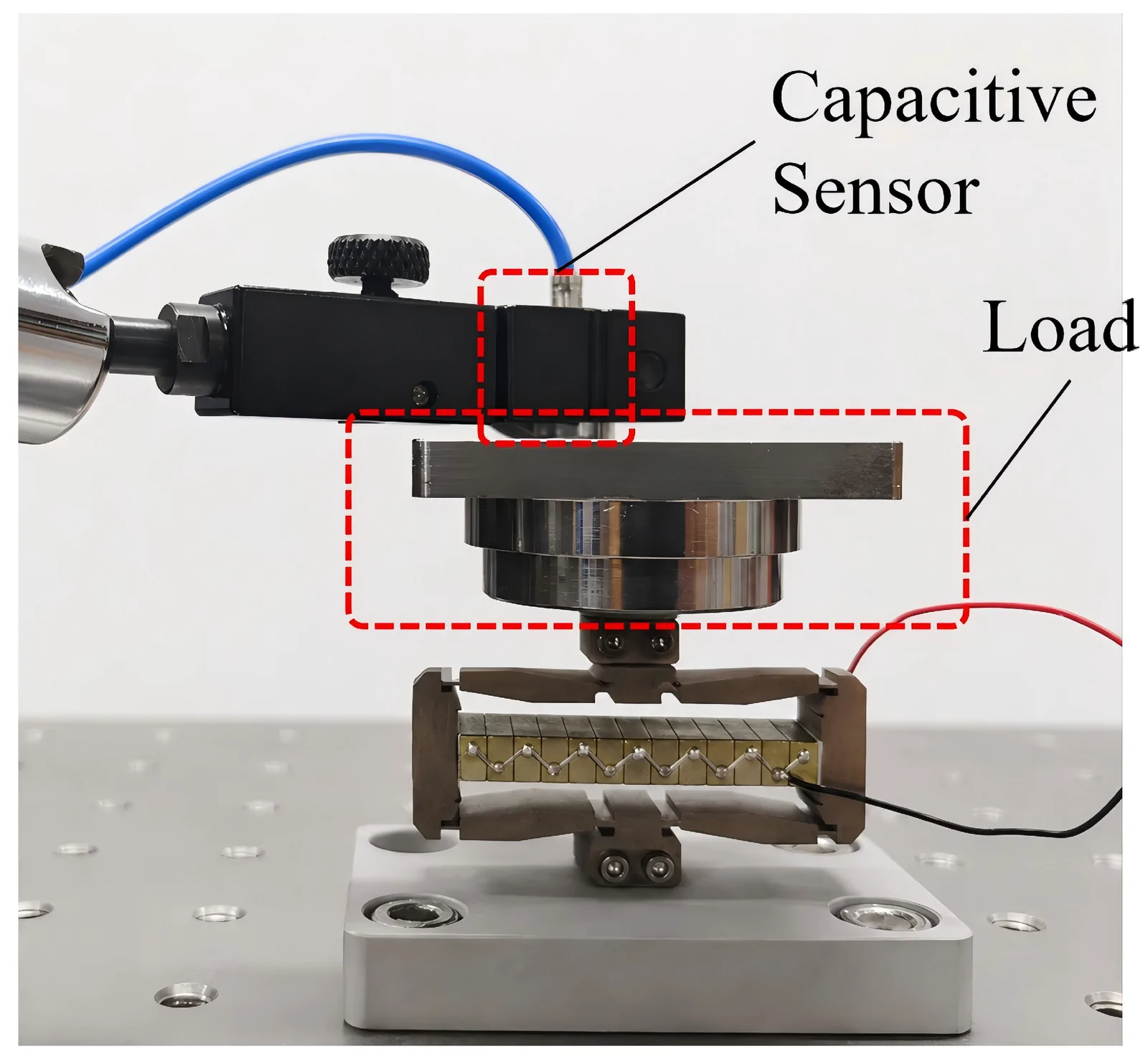
Amplification ratio and displacement loss test.

The PZT–amplification mechanism assembly is fixed at one end, and the output displacement at the other end is measured using a capacitive displacement sensor (Figure 16). The amplification ratio is calculated from the measured output displacement and the input displacement of the PZT. Different weights are then placed at the output end to validate the LDL model.

#### 5.1.4. Natural Frequency Measurement

**Figure 17:**
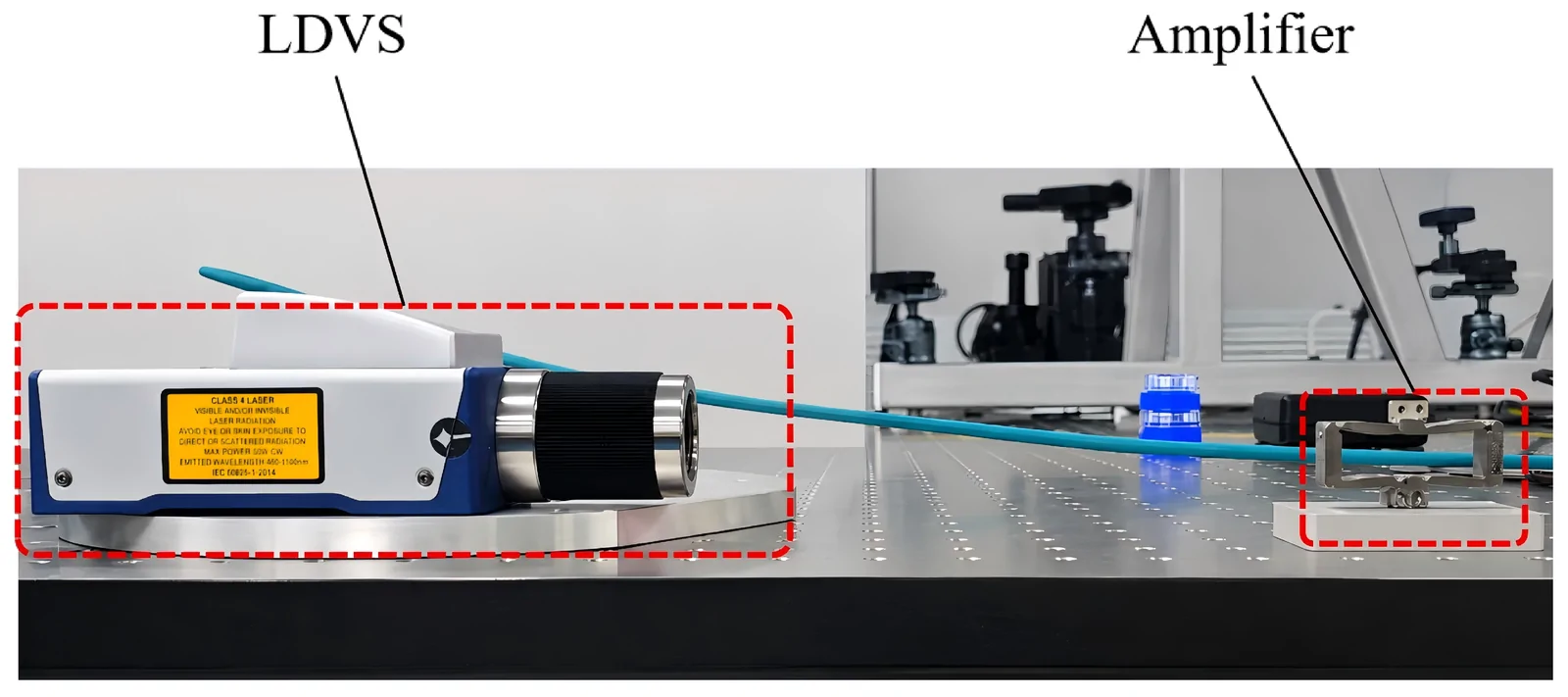
Natural frequency test.

The natural frequency of the amplification mechanism is measured using an impact test. One end of the amplification mechanism is fixed, and the other end is left free. The mechanism is excited by a metal hammer, and the vibration response is measured using a laser vibrometer (Polytec VFX-F-110, resolution: 0.1 Hz, Polytec GmbH, Waldbronn, Germany). The natural frequency is then extracted from the measured vibration response (Figure 17).

For the equivalent one-dimensional elastic member with length *L*, the natural frequency with one end fixed and the other end free is half of the first nonzero elastic frequency when both ends are free:

(30) $$\begin{array}{cc} f & =\frac{c}{4L},\\ f_{0} & =\frac{c}{2L}, \end{array}$$

where *c* is the wave velocity.

### 5.2. Experimental Results and Discussion

The experimental results of the main performance indices are summarized and compared with the theoretical predictions (Table 4).

**Figure 18:**
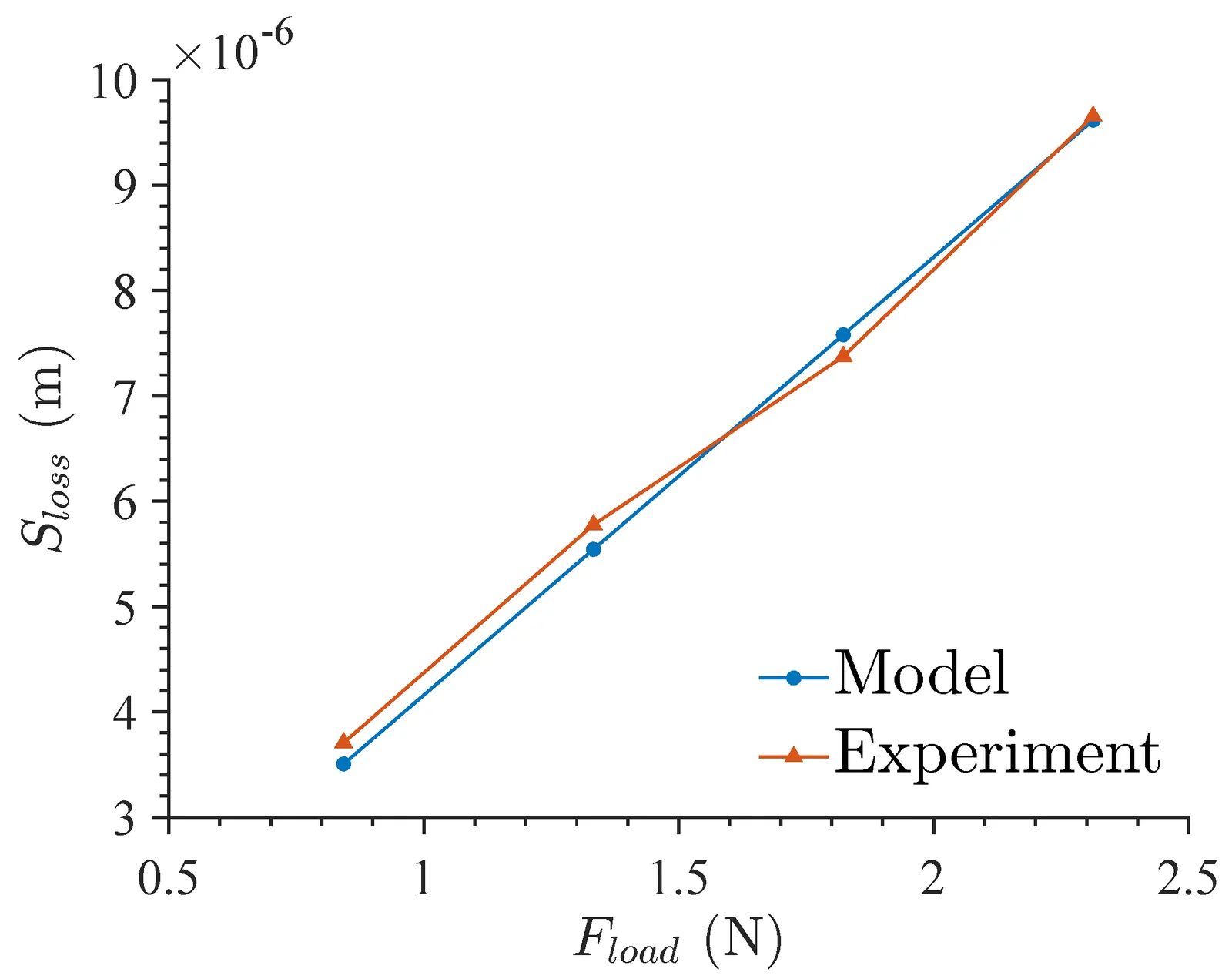
Comparison between LDL model predictions and experimental results.

The stiffness of the PZT stack is measured as 79 N/μm. This value is substituted into Equation (27) to calculate the theoretical displacement loss, which is then compared with the experimental results (Figure 18). The theoretical and experimental results show consistent trends, and the maximum error is 7.28%. This error mainly originates from measurement uncertainty caused by creep of the PZT output displacement [29]. These results indicate that the LDL model can adequately predict load-induced displacement loss.

The proposed model is compared with representative studies in the field of flexure amplification mechanism modeling (Table 5). Shi et al. [30] modeled elliptical flexure hinges using the compliance matrix method. Liu et al. [31] established a dynamic model using the Lagrange method. Wu et al. [32] modeled a rhombic amplification mechanism with elliptical beam flexures using the energy method. Compared with these models, the proposed model achieves comparable prediction accuracy for the amplification ratio and provides competitive prediction accuracy for output stiffness and fundamental frequency. Although the proposed model does not yield the lowest error for every individual index, it provides a unified framework for predicting amplification ratio, stiffness, fundamental frequency, and load-induced displacement loss, which is more suitable for design-oriented optimization. These results indicate that the proposed model has satisfactory prediction accuracy and can provide effective guidance for the design of flexure amplification mechanisms.

## 6. Conclusions

This study developed the MCH model for multi-configuration hinge analysis of flexure amplification mechanisms in PZT-actuated nanopositioning stages. The model addresses the high computational cost and limited prediction accuracy of existing modeling methods. By incorporating different flexure hinge configurations, the proposed model improves the prediction accuracy for key performance indices, providing an efficient tool for preliminary design, parameter screening, and structural optimization. The analysis shows that the V-shaped hinge can provide a higher stiffness upper limit than the other investigated hinge configurations. This finding indicates that the stiffness and performance envelope of flexure amplification mechanisms can be improved through hinge-configuration replacement, rather than only through geometric parameter adjustment.

The LDL model was also established to predict displacement loss under external loading and in systems coupled with other flexure mechanisms. The LDL model extends displacement prediction from the no-load condition to practical conditions involving preload, external payloads, guiding mechanisms, or multistage amplification structures. Finite element simulations and experiments show that the proposed models provide higher prediction accuracy than the conventional models considered in this study. The maximum prediction errors of the amplification ratio, output stiffness, fundamental frequency, and load-induced displacement loss are 4.87%, 4.92%, 7.77%, and 7.28%, respectively.

Based on the established models, sensitive design parameters were identified, and corresponding optimization rules were summarized. These rules can help researchers and designers determine the main design variables, avoid inefficient parameter combinations, and reduce the dependence on repeated finite element simulations during the early design stage. A single-stage amplification mechanism with a high amplification ratio of 17.55 was designed, validating the feasibility of the proposed design method. Overall, the proposed method provides a computationally efficient and experimentally validated modeling framework for designing flexure amplification mechanisms with large stroke, high load capacity, and favorable dynamic performance.

## Figures and Tables

**Table 1:** Finite element simulation parameters of the amplification mechanism.

Hinge Configuration	*l* (mm)	$d_{x}$ (mm)	*t* (mm)	$d_{y}$ (mm)	*R* (mm)	$h_{v}$ (mm)
Leaf	4	10	0.5	1	–	–
Filleted leaf	4	10	0.5	1	2	– ^1^
V-shaped	4	10	0.5	1	0.2 ^2^	2

^1^ “–” indicates that the corresponding parameter is not applicable to this hinge configuration. ^2^ This value denotes the fillet radius at the bottom of the V-shaped notch.

**Table 2:** Comparison between finite element simulations and theoretical results.

$d_{y}$	*A*			$K_{\mathrm{out}}$ (N/m)			σ (Pa)			*f* (Hz)		
(mm)	Analytical	FEM	Error	Analytical	FEM	Error	Analytical	FEM	Error	Analytical	FEM	Error
0.6	11.02	11.42	3.46%	$2.811\times 10^{4}$	$2.673\times 10^{4}$	4.92%	$4.00\times 10^{7}$	$4.17\times 10^{7}$	4.17%	162.31	150.14	7.50%
0.7	9.682	10.00	3.21%	$2.770\times 10^{4}$	$2.641\times 10^{4}$	4.63%	$4.60\times 10^{7}$	$4.81\times 10^{7}$	4.40%	160.84	149.53	7.03%
0.8	8.444	8.692	2.86%	$2.743\times 10^{4}$	$2.634\times 10^{4}$	3.99%	$5.20\times 10^{7}$	$5.40\times 10^{7}$	3.73%	159.77	152.56	4.51%
0.9	7.682	7.888	2.61%	$2.725\times 10^{4}$	$2.628\times 10^{4}$	3.56%	$5.80\times 10^{7}$	$6.05\times 10^{7}$	4.06%	158.95	153.37	3.51%
1.0	6.757	6.920	2.37%	$2.712\times 10^{4}$	$2.623\times 10^{4}$	3.28%	$6.20\times 10^{7}$	$6.46\times 10^{7}$	4.21%	158.28	148.23	6.35%

**Table 3:** Fixed parameters of the experimental amplification mechanisms.

$d_{x}$ (mm)	*l* (mm)	*R* (mm)	*w* (mm)	Material
17	2.2	0.4	10	316 stainless steel

**Table 4:** Comparison between experimental and theoretical results of the amplification mechanism.

*t*	$d_{y}$	*A*			$K_{\mathrm{out}}$ (N/m)			*f* (Hz)		
(mm)	(mm)	Theoretical	Experimental	Error	Theoretical	Experimental	Error	Theoretical	Experimental	Error
0.3	1.0	18.57	18.09	2.61%	$1.375\times 10^{4}$	$1.420\times 10^{4}$	3.25%	58.89	62.0	5.04%
0.4	1.0	18.13	17.54	3.22%	$3.260\times 10^{4}$	$3.334\times 10^{4}$	2.27%	90.67	96.0	5.58%
0.5	1.0	17.58	16.80	4.44%	$6.440\times 10^{4}$	$6.301\times 10^{4}$	3.74%	127.42	138.1	7.77%
0.6	1.0	16.97	16.42	3.22%	$1.135\times 10^{5}$	$1.187\times 10^{5}$	4.63%	169.19	160.7	5.30%
0.4	0.8	11.72	11.15	4.87%	$3.150\times 10^{4}$	$3.405\times 10^{4}$	4.67%	88.72	82.8	7.08%
0.4	1.2	7.918	7.494	2.83%	$3.110\times 10^{4}$	$3.263\times 10^{4}$	4.92%	87.66	82.5	6.26%

**Table 5:** Comparison of prediction accuracy with existing models.

Model	*A*		$K_{\mathrm{out}}$ (N/m)		*f* (Hz)	
	FEM Err.	Experimental Error	FEM Error	Experimental Error	FEM Error	Experimental Error
Proposed method	3.46%	4.87%	4.90%	4.92%	7.49%	7.77%
[30]	– ^1^	4.6%	–	9.9%	–	–
[31]	7.97%	14.1%	–	–	7.83%	11.0%
[32]	5.26%	2.97%	8.25%	6.25%	7.5%	3.5%

^1^ Not reported in the cited literature.

## Data Availability

The data presented in this study are available upon request from the corresponding author. Specific experimental data have been published in the article.

## Appendix A. Formula Derivations

### Appendix A.1. Compliance Factors of Three Configurations of Hinges

Using the thickness function t(x) and the design parameters defined in Figure 4, the factors in Equation (8) are obtained as follows.

- Leaf hinge:
  (A1) $$\begin{array}{cc} C_{11} & =\frac{l}{Ewt},\\ C_{22} & =\frac{4l^{3}}{Ewt^{3}},\\ C_{23} & =-\frac{6l^{2}}{Ewt^{3}},\\ C_{33} & =\frac{12l}{Ewt^{3}}. \end{array}$$
- Filleted leaf hinge:
  Local thickness function:
  (A2) $$t\left(x\right)=\left\{\begin{array}{cc} 2\left(R-\sqrt{2Rx-x^{2}}\right)+t,\; & 0\leq x<R,\\ t,\; & R\leq x<l-R,\\ 2\left(R-\sqrt{2R\left(l-x\right)-{\left(l-x\right)}^{2}}\right)+t,\; & l-R\leq x\leq l. \end{array}\right.$$
  Compliance factors:
  (A3) $$\begin{array}{cc} C_{11} & =\frac{1}{Ew}\left[\frac{l-2R}{t}+2\alpha t^{-1/2}\beta^{-1/2}\left(\arctan\left(\gamma^{1/2}\right)-\frac{\pi }{2}\right)\right]\\ C_{22} & =\frac{3}{Ew}[\frac{t^{1/2}\beta^{1/2}\left[-80R^{4}+24R^{3}t+8\left(3+2\pi \right)R^{2}t^{2}+4\left(1+2\pi \right)Rt^{3}+\pi t^{4}\right]}{4t^{5/2}\beta^{5/2}}\\ & \;+\frac{4\alpha^{3}\left(6R^{2}-4Rt-t^{2}\right)\arctan\left(\sqrt{\gamma }\right)}{4t^{5/2}\beta^{5/2}}\\ & -\frac{2\alpha \left[-24{\left(1-R\right)}^{2}R^{2}-8R^{3}t+14R^{2}t^{2}+8Rt^{3}+t^{4}\right]\arctan\left(\sqrt{\gamma }\right)}{t^{5/2}\beta^{5/2}}\\ & +\frac{4\left(l-2R\right)\left(t^{2}-tR+R^{2}\right)}{3t^{3}}\\ & +\frac{-40R^{4}+8lR^{2}\left(2R-t\right)+12R^{3}t+4\left(3+2\pi \right)R^{2}t^{2}+2\left(1+2\pi \right)Rt^{3}+\frac{1}{2}\pi t^{4}}{t^{2}\beta^{2}}\\ & +\frac{8l^{2}R\left(6R^{2}+4Rt+t^{2}\right)}{\alpha t^{2}\beta^{2}}]\\ C_{23} & =-\frac{6l}{Ew^{3}}\left[12R^{2}\alpha t^{1/2}\beta^{1/2}\arctan\left(\sqrt{\gamma }\right)+\alpha \beta^{2}-4R^{2}\left(16R^{2}+13Rt+3t^{2}\right)\right]\alpha^{-1}\beta^{-2}\\ C_{33} & =\frac{12}{Ewt^{3}}\left[l+2R\left[6R\beta^{1/2}\alpha^{2}\arctan\left(\sqrt{\gamma }\right)+t\beta \left(6R^{2}+4Rt+t^{2}\right)-1\right]\alpha^{-1}\beta^{-3}\right]. \end{array}$$
  Here, α=2R+t, β=4R+t, γ=1+4R/t.
- V-shaped hinge:
  For the V-shaped hinge, the cutting angle is defined as
  (A4) $$\theta_{v}=\arctan\left(\frac{2h_{v}}{l}\right).$$
  Local thickness function:
  (A5) $$t\left(x\right)=\left\{\begin{array}{cc} 2h_{v}-\frac{4h_{v}}{l}x+t,\; & 0\leq x<\frac{l}{2}-R\sin\theta_{v},\\ t_{\mathrm{arc}}\left(x\right),\; & \frac{l}{2}-R\sin\theta_{v}\leq x<\frac{l}{2}+R\sin\theta_{v},\\ \frac{4h_{v}}{l}x-2h_{v}+t,\; & \frac{l}{2}+R\sin\theta_{v}\leq x\leq l. \end{array}\right.$$
  Compliance factors:
  (A6) $$\begin{array}{cc} C_{11}\; & =\frac{1}{Ew}\left[\cot\theta \ln\left(\frac{\beta (1+\gamma )}{\gamma (1+\beta -\cos\theta )}\right)+\frac{2(1+\beta )}{\sqrt{2\beta +\beta^{2}}}\arctan\left(\sqrt{\frac{2+\beta }{\beta }}\tan\frac{\theta }{2}\right)-\theta \right],\\ C_{22}\; & =\frac{3}{2Ew}\frac{{\left[\gamma +(\beta -\gamma )\cos\theta \right]}^{2}}{\left(2\beta +\beta^{2}\right)\gamma^{2}\sin^{2}\theta }[\frac{(1+\beta )\sin\theta }{{\left(1+\beta -\cos\theta \right)}^{2}}+\frac{(3+2\beta +\beta^{2})\sin\theta }{\left(2\beta +\beta^{2}\right)\left(1+\beta -\cos\theta \right)}\\ & \;+\frac{6(1+\beta )}{{\left(2\beta +\beta^{2}\right)}^{3/2}}\arctan\left(\sqrt{\frac{2+\beta }{\beta }}\tan\frac{\theta }{2}\right)]\\ & \;-\frac{{\left[\gamma +(\beta -\gamma )\cos\theta \right]}^{2}}{\sin^{2}\theta }\left[\frac{2\cot\theta }{\beta^{2}{\left(1+\gamma \right)}^{2}}-\frac{\cot\theta }{\gamma^{2}{\left(1+\beta -\cos\theta \right)}^{2}}\right]\\ & \;-\frac{1+\beta }{{\left(1+\beta -\cos\theta \right)}^{2}}\left[\frac{3\beta^{2}+6\beta +1}{\left(\beta^{2}+2\beta \right)\left(1+\beta -\cos\theta \right)}\right]\sin\theta \\ & \;+\left[\frac{4(1+\beta )}{\sqrt{\beta^{2}+2\beta }}-\frac{2(1+\beta )}{{\left(2\beta +\beta^{2}\right)}^{3/2}}\right]\arctan\left(\sqrt{\frac{2+\beta }{\beta }}\tan\frac{\theta }{2}\right)+2\theta \\ & \;-2\left[\frac{\gamma +(\beta -\gamma )\cos\theta }{\beta (1+\gamma )\sin\theta }-\frac{\sin\theta }{1+\beta -\cos\theta }\right]\cot^{2}\theta +\frac{\sin^{2}\theta \cot\theta }{{\left(1+\beta -\cos\theta \right)}^{2}}\\ & \;+2\cot^{3}\theta \ln\left(\frac{\beta (1+\gamma )}{\gamma (1+\beta -\cos\theta )}\right),\\ C_{23}\; & =\frac{3\gamma +3(\beta -\gamma )\cos\theta }{2EwR\gamma \sin\theta }\frac{1}{2\beta +\beta^{2}}[\frac{(1+\beta )\sin\theta }{{\left(1+\beta -\cos\theta \right)}^{2}}+\frac{(3+2\beta +\beta^{2})\sin\theta }{\left(2\beta +\beta^{2}\right)\left(1+\beta -\cos\theta \right)}\\ & \;+\frac{6(1+\beta )}{{\left(2\beta +\beta^{2}\right)}^{3/2}}\arctan\left(\sqrt{\frac{2+\beta }{\beta }}\tan\frac{\theta }{2}\right)]\\ & \;-\frac{\gamma^{2}\cot\theta }{\beta^{2}{\left(1+\gamma \right)}^{2}}+\frac{\cot\theta }{{\left(1+\beta -\cos\theta \right)}^{2}},\\ C_{33}\; & =\frac{3}{2EwR^{2}\left(2\beta +\beta^{2}\right)}[\frac{(1+\beta )\sin\theta }{{\left(1+\beta -\cos\theta \right)}^{2}}+\frac{(3+2\beta +\beta^{2})\sin\theta }{\left(2\beta +\beta^{2}\right)\left(1+\beta -\cos\theta \right)}\\ & \;+\frac{6(1+\beta )}{{\left(2\beta +\beta^{2}\right)}^{3/2}}\arctan\left(\sqrt{\frac{2+\beta }{\beta }}\tan\frac{\theta }{2}\right)]\\ & \;-\frac{\gamma^{2}\cot\theta }{\beta^{2}{\left(1+\gamma \right)}^{2}}+\frac{\cot\theta }{{\left(1+\beta -\cos\theta \right)}^{2}}. \end{array}$$
  Here, β=t/2R, $\gamma =t/2h_{v}$, and $h_{v}$ is the cutting depth of the V-shaped notch.

### Appendix A.2. Stress Concentration Factors of Three Configurations of Hinges

- Leaf hinge:
  (A7) $$K_{a}=K_{b}=1$$
- Filleted leaf hinge:
  (A8) $$\begin{array}{cc} K_{a} & =3.065-3.370\left(\frac{2R}{2R+t}\right)+0.647{\left(\frac{2R}{2R+t}\right)}^{2}+0.658{\left(\frac{2R}{2R+t}\right)}^{3}\\ K_{b} & =3.065-6.269\left(\frac{2R}{2R+t}\right)+7.015{\left(\frac{2R}{2R+t}\right)}^{2}-2.812{\left(\frac{2R}{2R+t}\right)}^{3} \end{array}$$
- V-shaped hinge:
  (A9) $$\begin{array}{cc} K_{a} & =A_{1}+A_{2}\left(\frac{2R}{2R+t}\right)+A_{3}{\left(\frac{2R}{2R+t}\right)}^{2}+A_{4}{\left(\frac{2R}{2R+t}\right)}^{3}\\ K_{b} & =B_{1}+B_{2}\left(\frac{2R}{2R+t}\right)+B_{3}{\left(\frac{2R}{2R+t}\right)}^{2}+B_{4}{\left(\frac{2R}{2R+t}\right)}^{3} \end{array}$$

Here, $A_{i}$ and $B_{i}$ with i=1,2,3,4 are tabulated factors determined from the notch geometry [33].
